# Sodium Bicarbonate versus N-Acetylcysteine plus hydration versus hydration alone for preventing contrast-associated acute kidney injury: A single-center retrospective analysis with propensity score matching

**DOI:** 10.1371/journal.pone.0353461

**Published:** 2026-07-09

**Authors:** Ying-Huan Ma, Wei Wei, Nan Li, Yan Sun, Xiao-Xu Chen, Xiu-Ying Hao, Ai-Guo Xie

**Affiliations:** Department of Nephrology, The Air Force Hospital of Northern Theater PLA, Shenyang, Liaoning, China; Sichuan University, CHINA

## Abstract

**Background:**

Contrast-associated acute kidney injury (CA-AKI, historically termed contrast-induced nephropathy, CIN) is a leading cause of iatrogenic acute kidney injury (AKI) following iodinated contrast administration; yet the optimal preventive strategy remains controversial, especially in mild-to-moderate-risk patients.

**Methods:**

This single-center retrospective observational study was conducted in strict accordance with the Strengthening the Reporting of Observational Studies in Epidemiology (STROBE) guidelines. We consecutively screened adult patients undergoing contrast-enhanced computed tomography (CT) or angiography at a tertiary hospital in China between June 2022 and January 2025. After propensity score matching (PSM), 240 adult patients were included in the final analysis, allocated 1:1:1 into three groups: conventional hydration alone (control), hydration plus N-acetylcysteine (NAC), and hydration plus sodium bicarbonate. 1:1:1 nearest-neighbor PSM was performed to minimize confounding bias, with a caliper of 0.05. The primary endpoint was the incidence of CA-AKI within 72 hours after contrast exposure, defined per the 2024 European Society of Urogenital Radiology (ESUR) guidelines. Secondary endpoints included dynamic changes in renal function, renal replacement therapy (RRT) requirement, hospital stay duration, adverse events, and subgroup analyses by comorbidities and contrast modalities.

**Results:**

After PSM, 240 patients (80 per group) were included in the final analysis, with well-balanced baseline characteristics across groups (all standardized mean differences <0.1, all P > 0.05). The overall incidence of CA-AKI was 15.00% in the control group, 2.50% in the NAC group, and 7.50% in the sodium bicarbonate group. Hydration plus NAC significantly reduced CA-AKI risk compared with hydration alone (RR = 0.17, 95% CI: 0.04–0.74, Bonferroni-adjusted P = 0.004). Sodium bicarbonate showed a numerically lower CA-AKI incidence than control, but the difference did not reach statistical significance after correction (RR = 0.50, 95% CI: 0.19–1.31, adjusted P = 0.121). Repeated-measures ANOVA revealed significant group, time, and group × time interaction effects on serum creatinine (Scr), blood urea nitrogen (BUN), and estimated glomerular filtration rate (eGFR) (all P < 0.001), with the mildest renal function fluctuations in the NAC group. The renoprotective efficacy of NAC was consistent across contrast-enhanced CT and angiography modalities. Advanced age, comorbid diabetes, comorbid hypertension, higher baseline Scr, and lower baseline eGFR were independent risk factors for CA-AKI [5,6,28] (all P < 0.05). No patients required RRT in any group, with no significant difference in hospital stay duration or mild adverse event incidence across groups (all P > 0.05).

**Conclusions:**

For patients with eGFR ≥ 30 mL·min ⁻ ¹·(1.73 m² ⁻ ¹), hydration combined with high-dose intravenous NAC significantly reduces the short-term incidence of CA-AKI compared with hydration alone, with a favorable safety profile and consistent efficacy across contrast modalities. Hydration plus sodium bicarbonate is a safe alternative for patients intolerant to NAC. These findings are hypothesis-generating and require verification in large-sample, multicenter prospective trials.

## 1. Introduction

### 1.1. Background and rationale

With the widespread application of iodinated contrast media in contrast-enhanced computed tomography (CT) and angiographic procedures, contrast-associated acute kidney injury (CA-AKI) has emerged as the third leading cause of hospital-acquired AKI globally. Defined per the 2024 updated ESUR guidelines as an AKI occurring within 72 hours of iodinated contrast administration [1], characterized by a ≥ 25% relative increase or ≥44.2 μmol/L absolute elevation in Scr from baseline (excluding other etiologies of renal impairment), CA-AKI is associated with prolonged hospital stays, accelerated CKD progression, long-term RRT dependency, and increased all-cause mortality [2].

The incidence of CA-AKI varies widely across populations: it affects 1%–3% of individuals with normal renal function but rises to 30%–50% in high-risk cohorts with comorbid diabetes, hypertension, preexisting CKD, or heart failure [3,4]. Well-recognized independent risk factors for CA-AKI include advanced age, diabetes, hypertension, reduced eGFR, high contrast medium dosage, intra-arterial contrast administration, and concurrent use of nephrotoxic drugs. In Chinese patients with mild-to-moderate renal impairment, the baseline incidence of CA-AKI has unique population characteristics [5], which provides the basis for the inclusion criteria setting in this study.

Intravenous hydration remains the cornerstone of CA-AKI prophylaxis [6], mitigating injury via intravascular volume expansion, dilution of tubular contrast concentrations, and accelerated contrast excretion. However, accumulating evidence shows that conventional hydration alone offers limited renal protection in high-risk patients [7], prompting extensive research into combined strategies with nephroprotective agents to optimize prophylaxis.

### 1.2. Mechanisms of investigated agents

The pathogenesis of CA-AKI is multifactorial, driven by interactions between renal medullary ischemia-hypoxia [8], oxidative stress [9,10], tubular epithelial cell injury [11], and intratubular obstruction. Iodinated contrast media trigger renal vasoconstriction, reducing medullary perfusion and inducing ischemia-hypoxia, while simultaneously stimulating excessive reactive oxygen species (ROS) production, which disrupts the redox balance and causes tubular cell apoptosis and necrosis [10]. Contrast media also form crystals in acidic tubular urine, leading to intratubular obstruction and further renal damage [11].

N-acetylcysteine (NAC), a sulfhydryl-containing antioxidant, exerts renoprotective effects via multiple targeted mechanisms: direct ROS scavenging, augmentation of endogenous glutathione synthesis to enhance antioxidant capacity [12], and renal vasodilation to improve medullary perfusion and mitigate ischemia-hypoxia injury [13]. Sodium bicarbonate prevents CA-AKI primarily through urinary alkalinization (target pH 7.0–8.0) [1,14], which reduces contrast media crystallization and tubular deposition, alleviates intratubular obstruction, and indirectly inhibits ROS production in renal tissue [15,16].

### 1.3. Research gap and objective

Despite extensive investigation, the prophylactic efficacy of NAC and sodium bicarbonate against CA-AKI remains highly controversial. The landmark PRESERVE trial (2018) [17], a multicenter randomized controlled trial (RCT), failed to demonstrate that either agent reduced 90-day severe renal adverse events in high-risk patients with eGFR < 60 mL·min ⁻ ¹·(1.73 m²)⁻¹. A subsequent reanalysis of the PRESERVE trial further clarified the reasons for the inconsistent conclusions [18], which are likely attributed to variations in study populations, intervention regimens, outcome metrics, and follow-up durations. Notably, most prior studies either compared a single agent to hydration alone or lacked robust confounding adjustment in retrospective designs, with few head-to-head comparisons of NAC and sodium bicarbonate in mild-to-moderate risk patients with eGFR ≥ 30 mL·min ⁻ ¹·(1.73 m²)⁻¹ [19].

Meta-analyses have confirmed that high-dose NAC has better renoprotective effects than low-dose NAC [20], and intravenous administration is superior to oral administration [21], but there is a lack of sufficient evidence in Chinese populations [22]. In addition, dose-response meta-analyses have confirmed that contrast medium doses >1.5 mL/kg significantly increase CA-AKI risk [23], which is an important basis for the control of contrast medium dosage in this study.

This single-center retrospective study was designed to compare the prophylactic efficacy and safety profiles of conventional hydration alone, hydration plus NAC, and hydration plus sodium bicarbonate for CA-AKI prevention. We employed 1:1:1 PSM to minimize confounding bias from non-randomized grouping, and performed stratified subgroup analyses by comorbidities (diabetes/hypertension) and contrast modalities (contrast-enhanced CT/angiography) to explore personalized efficacy. The study was conducted at the Air Force Hospital of the Northern Theater PLA, a tertiary general hospital with an annual volume of ~3,000 contrast-enhanced CT/angiography procedures, with all patients managed per a standardized institutional clinical protocol for CA-AKI prophylaxis formulated in 2022. We aimed to provide evidence-based recommendations for clinical CA-AKI prophylaxis in mild-to-moderate-risk patients with preserved or mildly impaired renal function [24].

## 2. Materials and methods

This study was reported in strict accordance with the Strengthening the Reporting of Observational Studies in Epidemiology (STROBE) guidelines [25].

### 2.1. Ethical approval and informed consent

This study was conducted in strict accordance with the Declaration of Helsinki (2024 revision) for medical research involving human subjects. The study protocol was approved by the Institutional Ethics Committee of the Air Force Hospital of the Northern Theater Command (Approval No.: 2025-Dept-014; Approval Date: May 15, 2025), with a written confirmation for retrospective data utilization (Confirmation No.: 2025-Valid-014).

Given the retrospective, non-interventional study design, the informed consent procedure was approved by the ethics committee with a standardized two-step strategy:

1. Written informed consent was obtained from all follow-up patients (successfully contacted via telephone or medical record review within 3 months post-examination, with complete post-contrast renal function data) before their retrospective clinical data were included in the study.
2. For patients lost to follow-up (failed to contact for >3 months or with incomplete post-contrast renal function test data), the requirement for informed consent was formally waived with written approval from the ethics committee (Approval Document No.: 2025-Exempt-003). The waiver of informed consent was publicly announced on the hospital’s official website for 1 month, with no objections received from the public or patients’ families.

All clinical data were fully anonymized by removing all personal identifiers to protect patient privacy, and only adult patients aged ≥18 years were enrolled without the need for parental/guardian consent. This study strictly followed the waiver clauses of the Declaration of Helsinki for non-interventional retrospective research, and the anonymized data utilization did not cause any harm to the rights, health, or privacy of the included patients.

### 2.2. Study design and setting

This was a single-center retrospective observational study conducted at the Department of Nephrology and Radiology, Air Force Hospital of Northern Theater PLA, a tertiary care hospital in Shenyang, China. The study period was from June 2022 to May 2025, with the follow-up endpoint set at 72 hours after contrast administration, consistent with the ESUR diagnostic time window for CA-AKI [1,26]. All patients received standardized clinical management in line with the hospital’s 2022 clinical practice protocol for contrast-enhanced imaging and CA-AKI prophylaxis [14].

### 2.3. Study population

We consecutively screened adult patients who underwent iodinated non-ionic contrast-enhanced CT (head and neck, chest, abdomen, pelvis) or angiography (coronary, peripheral) at our hospital during the study period.

#### 2.3.1. Inclusion criteria.

1. Aged ≥18 years old, underwent contrast-enhanced CT or angiography with non-ionic low-osmolar iodinated contrast media (iohexol injection, 300 mgI/mL);
2. Complete clinical data are available, including baseline demographic characteristics, medical history, liver and kidney function parameters, electrolyte levels, echocardiographic results, medication records, and contrast medium dosage.
3. Renal function test results (Scr, BUN) are available at baseline (within 24 hours before contrast administration) and at 24 h, 48 h, and 72 h after contrast exposure [1,26];
4. Baseline eGFR ≥ 30 mL·min ⁻ ¹·(1.73 m ⁻ ²), calculated using the validated Modification of Diet in Renal Disease (MDRD) equation [27].

#### 2.3.2. Exclusion criteria.

1. History of hypersensitivity or allergy to iodinated contrast media, NAC, or sodium bicarbonate;
2. Severe hepatic dysfunction: alanine aminotransferase (ALT) or aspartate aminotransferase (AST) >2 × the upper limit of normal (ULN), or total bilirubin >1.5 × the ULN;
3. Congestive heart failure with NYHA classification Ⅲ–Ⅳ, or severe electrolyte disturbances (serum potassium <3.5 mmol/L or >5.5 mmol/L);
4. Use of nephrotoxic drugs (e.g., aminoglycoside antibiotics, non-steroidal anti-inflammatory drugs) or exposure to iodinated contrast media within 7 days before the index examination;
5. Pregnant or lactating women (confirmed via a pregnancy test or medical history);
6. Comorbidities that may interfere with renal function assessment, including stage Ⅳ advanced malignant tumors, sepsis, or congenital/acquired coagulation disorders;
7. Incomplete clinical data or lost to follow-up with unavailable post-contrast renal function test results.

#### 2.3.3. Rationale for Excluding Patients with eGFR < 30 mL·min ⁻ ¹·(1.73 m²)⁻¹.

1. Clinical practice: Patients with eGFR < 30 mL·min ⁻ ¹·(1.73 m²)⁻¹ (severe CKD) receive a specialized institutional CA-AKI prophylaxis regimen (low-dose contrast, prolonged hydration, individualized drug selection) that is not comparable to the regimens evaluated in this study [28,29];
2. Statistical validity: This population has an extremely high CA-AKI incidence (50%), which would introduce significant selection bias and mask the protective efficacy of NAC and sodium bicarbonate in mild-to-moderate-risk patients [3].
3. Safety evaluation: Sodium bicarbonate use in patients with severe renal insufficiency is associated with a high risk of electrolyte disturbances (hypernatremia, metabolic alkalosis) [30], which would confound the safety assessment of the study regimens.

### 2.4. Group allocation

Group allocation was based on the standardized 2022 institutional clinical practice protocol for CA-AKI prophylaxis [14], rather than subjective attending physician judgment, to minimize physician-related selection bias. The protocol was uniformly implemented by all clinicians in the department, with clear, objective criteria for assigning patients to each group:

**Control group (hydration alone):** Low-risk patients with no diabetes/hypertension, eGFR ≥ 90 mL·min ⁻ ¹·(1.73 m²)⁻¹, and contrast medium dosage <100 mL (median: 92.5 mL, IQR: 85.0–98.0 mL); this cohort included patients with potential renal susceptibility factors (age ≥ 50 years in 68.75% of cases, and underlying cardiovascular disease in 18.75% of cases).

**NAC group:** Mild-to-moderate risk patients with 1–2 high-risk factors (diabetes, hypertension, eGFR 30–89 mL·min ⁻ ¹·(1.73 m²)⁻¹).

**Sodium bicarbonate group:** Mild-to-moderate risk patients with contraindications/intolerance to NAC, or who refused NAC treatment.

To further eliminate the potential confounding bias from risk-stratified grouping, we included all core baseline risk factors (age, gender, comorbidities, baseline eGFR, contrast medium dosage, and NYHA classification) into the 1:1:1 PSM model to ensure balanced baseline characteristics across the three groups after matching.

### 2.5. Propensity Score Matching (PSM)

To minimize residual confounding bias from non-randomized grouping, we performed 1:1:1 nearest-neighbor PSM with a caliper of 0.05 using SPSS 26.0 software.

1. Propensity score model: A multinomial logistic regression model was used to estimate the propensity score for each patient, with group allocation as the dependent variable.
2. Matching covariates: Pre-specified matching variables included age, gender, comorbidities (hypertension, diabetes, coronary heart disease), baseline eGFR, contrast medium dosage, and NYHA cardiac function classification.
3. Matching algorithm: 1:1:1 nearest-neighbor matching without replacement was performed, with a caliper width of 0.05 of the standard deviation of the logit of the propensity score.
4. Balance assessment: Standardized mean difference (SMD) was used to evaluate intergroup balance before and after matching, with SMD < 0.1 indicating excellent comparability. We also compared the distribution of propensity scores before and after matching to confirm matching validity, and verified that all matched samples were within the common support domain with no extreme outliers.

A total of 326 eligible patients were included in the PSM model, and 240 patients (80 per group) were successfully matched with a 100% matching success rate (no samples were excluded due to exceeding the caliper range). Before matching, several baseline covariates showed significant intergroup differences (SMD > 0.1, P < 0.05); after matching, all covariates had SMD < 0.1 and P > 0.05, confirming excellent intergroup balance.

### 2.6. Sample size calculation

The minimum required sample size was calculated a priori based on the primary outcome (incidence of CA-AKI). We assumed a 15% CA-AKI incidence in the control group (consistent with Chinese population data [24]) and a 5% incidence in the NAC intervention group. With a two-tailed α = 0.05 and 80% statistical power, the minimum required sample size was 76 patients per group. We rounded up to 80 patients per group to account for potential minor data inconsistencies.

No attrition rate was incorporated into the sample size calculation, as the retrospective study design with strict inclusion criteria (complete baseline and 72-hour follow-up renal function data) ensured all enrolled patients had intact primary outcome data.

Post-hoc power analysis was performed using G*Power 3.1 software to confirm the reliability of the results:

For the primary outcome comparison between the control and NAC groups: The observed effect size (w = 0.32) yielded a post-hoc statistical power of 86.2%, exceeding the pre-specified 80% threshold.

For subgroup analyses: The statistical power was 72% for the diabetes subgroup and 74% for the hypertension subgroup, both below 80%, which is acknowledged as a limitation in Section 4.6.

For repeated-measures ANOVA of renal function indices: The post-hoc power was > 90% for all group × time interaction effects.

The minimum required sample size was 80 patients per group for the primary outcome with a two-tailed α = 0.05 and 80% statistical power. No attrition rate was incorporated into the sample size calculation, as the retrospective study design with strict inclusion criteria (complete clinical and follow-up data) ensured all enrolled patients had intact primary outcome data.

### 2.7. Treatment protocols

All patients received standardized basic clinical care, including strict blood pressure control (target: < 140/90 mmHg for non-diabetic patients, < 130/80 mmHg for diabetic patients), fasting for 4–6 hours before the examination, and discontinuation of nephrotoxic drugs 7 days before contrast exposure. A uniform non-ionic low-osmolar iodinated contrast medium (iohexol injection, 300 mgI/mL) was used for all patients [14], with dosage determined by the examination site, procedural requirements, and patient body weight (range: 1.5–2.0 mL/kg) [23]. Actual contrast medium dosage and patient body weight were recorded in detail in the hospital’s electronic medical record system (EMRS).

#### 2.7.1. Standardized hydration protocol.

All patients received a standardized hydration protocol initiated 12 hours before contrast administration and continued for 24 hours after exposure [6]. For the control group and NAC group, the hydration solution was 0.9% sodium chloride injection, infused intravenously at a rate of 1 mL·kg ⁻ ¹·h ⁻ ¹, with a total infusion volume of no less than 1500 mL. For the sodium bicarbonate group, the hydration solution was combined with sodium bicarbonate for alkalization, and the infusion volume and rate were calibrated to control the total sodium load. For patients with NYHA class Ⅰ–Ⅱ cardiac function, the infusion rate was adjusted based on urine output and hemodynamic status to prevent volume overload [31], with continuous monitoring of heart rate, blood pressure, and urine output during infusion. The total sodium load of all three groups was standardized and controlled within a clinically equivalent range (the difference in total sodium intake was < 5 mmol/kg), and sodium concentration was not considered a confounding factor in the study due to the negligible clinical impact of this minor difference.

#### 2.7.2. NAC intervention.

On top of standardized hydration, patients in the NAC group received an intravenous infusion of NAC injection (20 mL: 4 g) 1 hour before contrast exposure, with a repeat dose 12 hours after exposure [32]. Each 1.2 g dose of NAC was dissolved in 250 mL of 0.9% sodium chloride injection, infused at a rate of 30 drops per minute.

**Rationale for the 12 g High-Dose Intravenous NAC Regimen:** This regimen is based on the latest clinical guidelines and meta-analyses [20,21], which confirm that high-dose intravenous NAC has superior renoprotective efficacy compared with low-dose oral NAC in high-risk patients. Intravenous administration avoids the first-pass effect of oral dosing [21], achieving higher, more rapid, and stable plasma drug concentrations to ensure sufficient sulfhydryl groups for ROS scavenging and glutathione synthesis [12]. This regimen has been the standard clinical practice at our hospital since 2022, with a well-documented safety profile in over 1,000 patients [22].

#### 2.7.3. Sodium bicarbonate intervention.

On top of standardized hydration, patients in the sodium bicarbonate group received an alkalizing mixture (150 mL of 5% sodium bicarbonate solution + 350 mL of 0.9% sodium chloride injection), infused intravenously at 40 drops per minute starting 1 hour before contrast administration and continuing for 24 hours after exposure [1,14].

Urine pH was measured every 2 hours using a portable urine pH meter throughout the intervention period, with all monitoring data recorded in the EMRS. The infusion rate and dosage were adjusted to maintain urine pH within the target range of 7.0–8.0 [1,14]:

1. If urine pH < 7.0: Infusion rate increased to 50 drops per minute, with a single additional 20 mL dose of 5% sodium bicarbonate injection;
2. If urine pH > 8.0: the infusion rate is reduced to 30 drops per minute.

More than 95% of patients in this group maintained stable urine pH within the target range throughout the intervention period.

### 2.8. Outcome measures

#### 2.8.1. Primary outcome.

The primary outcome was the incidence of CA-AKI within 72 hours after contrast administration, diagnosed in strict accordance with the 2024 ESUR Clinical Practice Guideline [1]: an increase in Scr of ≥25% relative to baseline, or an absolute increase of ≥44.2 μmol/L, within 72 hours of contrast exposure, after excluding other etiologies of renal impairment (nephrotoxic drugs, hypoperfusion, severe infection, urinary tract obstruction). CA-AKI diagnosis was independently adjudicated by two nephrologists who were blinded to the patients’ group allocation, with discrepancies resolved by a third senior chief physician.

#### 2.8.2. Secondary outcomes.

1. Dynamic changes in renal function indices (Scr, BUN, eGFR) at baseline, 24 h, 48 h, and 72 h after contrast exposure [26];
2. Clinical outcomes: requirement for RRT due to CA-AKI-induced severe AKI, and total hospital stay duration (defined as the interval from admission to discharge for the index contrast-enhanced examination);
3. Incidence and severity of adverse events during the intervention period [30];
4. Incidence of CA-AKI in stratified subgroups:
  - By underlying comorbidities: diabetes/non-diabetes, hypertension/non-hypertension [3,4];
  - By contrast, administration modality: contrast-enhanced CT (intravenous) vs. angiography (intra-arterial) [33].

#### 2.8.3. Laboratory measurements.

Five milliliters of peripheral venous blood were collected from patients at baseline and 24 h, 48 h, and 72 h after contrast exposure. Samples were centrifuged at 3000 r/min for 10 minutes to separate serum, and Scr and BUN levels were measured using a Beckman AU5800 automatic biochemical analyzer. eGFR was calculated using the MDRD equation [27], validated for short-term renal function assessment in AKI patients [26].

**Rationale for Fixed Time Point Blood Collection:** This blood collection schedule is part of the routine clinical care protocol for all contrast-enhanced imaging patients at our hospital, mandated per ESUR and KDIGO guidelines for serial renal function monitoring to detect early AKI [1,26]. All test results were prospectively collected and stored in the EMRS for clinical decision-making, and were retrospectively extracted for this study. In addition, novel AKI biomarkers such as neutrophil gelatinase-associated lipocalin (NGAL) and kidney injury molecule-1 (KIM-1) have better early diagnostic value [34], which is a limitation of this study for not including relevant detection.

#### 2.8.4. Baseline data collection.

Comprehensive baseline clinical data were extracted from the EMRS, including:

- Demographic characteristics: age, gender, body weight;
- Clinical history: hypertension, diabetes, coronary heart disease, NYHA cardiac function classification, and LVEF (via echocardiography) [3,4];
- Baseline laboratory results: liver and kidney function indices, electrolyte levels;
- Baseline medication use: ACEi/ARB, beta-blockers, diuretics, statins, antioxidants [35];
- Procedural data: contrast medium dosage [23], contrast administration modality [33];
- CKD stage, classified per the KDIGO 2023 criteria [3]: Stage 1 (eGFR ≥ 90 mL·min ⁻ ¹·(1.73 m² ⁻ ¹)), Stage 2 (eGFR 60–89 mL·min ⁻ ¹·(1.73 m² ⁻ ¹)), Stage 3 (eGFR 30–59 mL·min ⁻ ¹·(1.73 m² ⁻ ¹)), Stages 4–5 (eGFR < 30 mL·min ⁻ ¹·(1.73 m² ⁻ ¹)).

#### 2.8.5. Adverse event assessment.

Adverse events during the intervention period (nausea, headache, rash, and electrolyte disturbances) were extracted from the EMRS [30], with severity graded as:

1. Mild: Resolved spontaneously without treatment;
2. Moderate: Required symptomatic treatment but did not interrupt the examination/treatment;
3. Severe: Required drug withdrawal or interruption of the examination/treatment.

### 2.9. Statistical methods

All data were collected and sorted using Excel 2019, with statistical analysis performed using SPSS 26.0 software. All statistical tests were two-tailed, with a significance level of α = 0.05.

1. Normality and homogeneity testing: The Shapiro-Wilk test was used to verify normality of continuous data, and the Levene test to verify homogeneity of variance.
2. Data presentation: Normally distributed continuous data with homogeneous variance are presented as the mean ± standard deviation (x̄ ± s); non-normally distributed continuous data are presented as the median (interquartile range) [M (IQR)], with 95% confidence intervals (95% CI). Categorical data are presented as numbers and percentages [n (%)].
3. Intergroup comparisons: For normally distributed continuous data across three groups, one-way ANOVA was used for homogeneous variance, with Welch’s ANOVA applied for variance heterogeneity. Post-hoc pairwise comparisons were performed using Bonferroni’s test, with a corrected α’ = 0.0167 (0.05/3 pairwise comparisons) to control for type I error. For categorical data, Pearson’s chi-square test was used, with Fisher’s exact test applied for theoretical frequencies <5.
4. Intragroup temporal comparisons: Repeated-measures ANOVA was used to compare renal function indices across time points (baseline, 24 h, 48 h, 72 h) within each group. Mauchly’s sphericity test was performed prior to analysis; the Greenhouse-Geisser correction was applied for violated sphericity assumptions (P < 0.05). When a significant group×time interaction effect was detected, simple effect analysis was performed to further explore the intergroup differences at each time point and intragroup differences between each pair of time points. Bonferroni correction was used for all pairwise comparisons in simple effect analysis (corrected α’ = 0.0167). Partial η² was used to represent the effect size, with partial η² > 0.138 indicating a large effect size, 0.059–0.138 a medium effect size, and <0.059 a small effect size.
5. Primary outcome analysis: The relative risk (RR) of CA-AKI was calculated using a modified Poisson regression model with robust error variance, with a 95% CI reported. Bonferroni correction was applied for pairwise group comparisons (corrected α’ = 0.0167).
6. Subgroup analysis: Fisher’s exact test was used to compare CA-AKI incidence across subgroups, with Bonferroni correction for multiple testing (corrected α’ = 0.0167). The interaction effect between subgroup variables and treatment efficacy was assessed via the interaction term in the multivariable logistic regression model, with P < 0.05 indicating a statistically significant interaction.
7. Multivariable regression analysis: Binary logistic regression with backward stepwise selection was used to identify independent risk factors for CA-AKI [5,6,28]. The dependent variable was CA-AKI occurrence (yes = 1, no = 0), with independent variables including intervention group (dummy variable: control group as reference, NAC group and sodium bicarbonate group as dummy variables), age, gender, comorbidities, baseline renal function indices, contrast medium dosage, NYHA classification, and baseline medication use. Variable entry criteria were set at P < 0.05, with removal at P > 0.1 to reduce multicollinearity. The variance inflation factor (VIF) was calculated to test for multicollinearity, with VIF < 5 indicating no significant multicollinearity.
8. Missing data handling: All included patients had complete primary outcome data; missing data for secondary outcomes were handled via complete-case analysis, with no imputation performed.
9. Post-hoc power analysis: Post-hoc statistical power was calculated using G*Power 3.1 software for all primary and secondary analyses, including subgroup analyses, to confirm the reliability of the results.

### 2.10. Data availability statement

The clinical data supporting the findings of this study are stored in the EMRS of Air Force Hospital of Northern Theater Command and are protected by hospital medical record management regulations and the Personal Information Protection Law of the People’s Republic of China, thus are not publicly available. De-identified individual participant data can be made available upon reasonable request to the corresponding authors, with formal written approval from the Institutional Ethics Committee of Air Force Hospital of Northern Theater Command. All requests must include a detailed research proposal stating the intended use of the data, a data management plan, and a signed data use agreement. Eligible requests will be responded to within 14 working days of receipt.

## 3. Results

### 3.1. Patient screening and baseline characteristics

**Fig 1:**
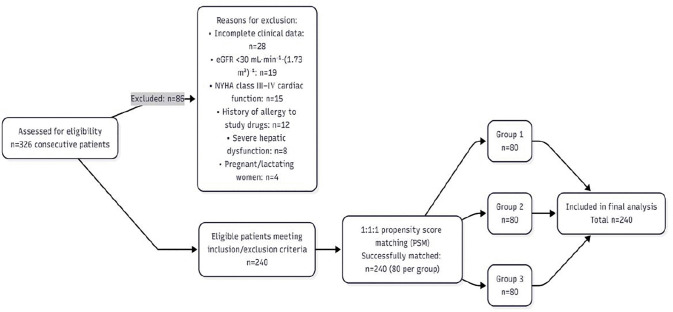
Flow chart of patient enrollment, screening, exclusion criteria, propensity score matching (PSM) process, and final included patients in the study.

A total of 387 consecutive patients were initially screened, with 61 patients excluded per the inclusion/exclusion criteria (28 with incomplete clinical data, 19 with eGFR < 30 mL·min ⁻ ¹·(1.73 m²)⁻¹, 15 with NYHA class Ⅲ–Ⅳ cardiac function, 12 with a history of allergy to study drugs, 8 with severe hepatic dysfunction, 4 pregnant/lactating women). A total of 326 eligible patients were included in the PSM model, and all 240 matched patients (80 per group) completed the 72-hour follow-up with full primary outcome data available, resulting in a 0% attrition rate(Fig 1).

After PSM, baseline characteristics were well balanced across the three groups, with no statistically significant differences in age, gender, body weight, contrast medium dosage, LVEF, baseline renal/liver function, electrolyte levels, comorbidity incidence, CKD stage distribution, or baseline medication use (all P > 0.05). The SMD of all baseline indicators was < 0.1 after matching, confirming excellent intergroup comparability (Table 1). The majority of patients were in CKD Stage 1 (72.08%), 25.42% in Stage 2, and 2.50% in Stage 3; no patients were in CKD Stages 4–5, consistent with the study’s inclusion criteria [15].

**Table 1:** Baseline characteristics of the three groups after PSM (n = 80 per group).

Index	Control group	NAC group	Sodium bicarbonate group	P value	SMD (after PSM)
**Continuous variables** **(x̄ ± s)**					
Age (years)	52.3 ± 10.5	53.1 ± 11.2	51.8 ± 10.8	0.879	<0.08
Weight (kg)	65.2 ± 8.3	64.8 ± 7.9	65.5 ± 8.1	0.912	<0.06
Contrast medium dosage (mL)	98.5 ± 15.2	97.8 ± 14.8	98.2 ± 15.0	0.935	<0.05
Scr (μmol/L)	78.5 ± 10.2	77.8 ± 10.5	78.2 ± 10.3	0.927	<0.07
BUN (mmol/L)	5.2 ± 1.1	5.1 ± 1.0	5.3 ± 1.1	0.858	<0.06
eGFR (mL·min ⁻ ¹·(1.73 m²)⁻¹)	95.6 ± 11.2	96.2 ± 11.5	95.8 ± 11.3	0.910	<0.08
LVEF (%)	62.5 ± 5.2	63.0 ± 4.9	62.8 ± 5.1	0.918	<0.05
**Categorical variables [n(%)]**					
Male	43 (53.75)	40 (50.00)	44 (55.00)	0.890	<0.07
Hypertension	30 (37.50)	33 (41.25)	29 (36.25)	0.842	<0.06
Diabetes	20 (25.00)	17 (21.25)	21 (26.25)	0.810	<0.08
Coronary heart disease	13 (16.25)	11 (13.75)	12 (15.00)	0.903	<0.05
NYHA Ⅰ	64 (80.00)	67 (83.75)	65 (81.25)	0.815	<0.06
NYHA Ⅱ	16 (20.00)	13 (16.25)	15 (18.75)	0.815	<0.06
CKD Stage 1	58 (72.50)	60 (75.00)	57 (71.25)	0.910	<0.05
CKD Stage 2	20 (25.00)	18 (22.50)	21 (26.25)	0.904	<0.05
CKD Stage 3	2 (2.50)	2 (2.50)	2 (2.50)	1.000	<0.01
ACEi/ARB use	23 (28.75)	24 (30.00)	22 (27.50)	0.957	<0.04
Beta-blocker use	18 (22.50)	19 (23.75)	17 (21.25)	0.950	<0.05
Diuretic use	8 (10.00)	7 (8.75)	9 (11.25)	0.898	<0.06
Statin use	31 (38.75)	33 (41.25)	30 (37.50)	0.950	<0.04

Note: SMD = standardized mean difference; CKD = chronic kidney disease; ACEi/ARB = angiotensin-converting enzyme inhibitor/angiotensin receptor blocker; LVEF = left ventricular ejection fraction; NYHA = New York Heart Association. P values were calculated via one-way ANOVA for continuous variables and Pearson’s chi-square test for categorical variables. SMD < 0.1 indicates excellent intergroup balance.

### 3.2. Dynamic changes in renal function indices

#### 3.2.1. Group, time, and interaction effects.

Mauchly’s sphericity test showed that the sphericity assumption was violated for Scr (χ² = 32.64, P < 0.001), BUN (χ² = 28.91, P < 0.001), and eGFR (χ² = 35.72, P < 0.001). Repeated-measures ANOVA with Greenhouse-Geisser correction revealed significant main effects of group and time, as well as significant group × time interaction effects on Scr, BUN, and eGFR (all P < 0.001). This indicates that renal function indices changed significantly over time after contrast administration in all groups, with significantly different change trajectories across the three groups. The group × time interaction effects for Scr and eGFR had large effect sizes (partial η² = 0.172 and 0.189, respectively), while the interaction effect for BUN had a medium effect size (partial η² = 0.073) (Table 2).

**Table 2:** Repeated-measures ANOVA results of renal function indices (after Greenhouse-Geisser correction).

Index	Source of variation	F value	P value	Partial η² (Effect size)
Scr	Group (Main effect)	20.145	<0.001	0.238
	Time point (Main effect)	115.268	<0.001	0.441
	Group × Time point (Interaction effect)	19.632	<0.001	0.172
BUN	Group (Main effect)	14.876	<0.001	0.140
	Time point (Main effect)	91.354	<0.001	0.382
	Group × Time point (Interaction effect)	6.943	<0.001	0.073
eGFR	Group (Main effect)	31.869	<0.001	0.265
	Time point (Main effect)	126.874	<0.001	0.476
	Group × Time point (Interaction effect)	21.357	<0.001	0.189

Note: All results were adjusted using the Greenhouse-Geisser correction due to violation of the sphericity assumption (Mauchly’s test, all P < 0.001). Partial η² is used to represent effect size: partial η² < 0.059 indicates a small effect size, 0.059–0.138 indicates a medium effect size, and >0.138 indicates a large effect size. Scr = serum creatinine; BUN = blood urea nitrogen; eGFR = estimated glomerular filtration rate.

#### 3.2.2. Simple effect analysis.

**Fig 2:**
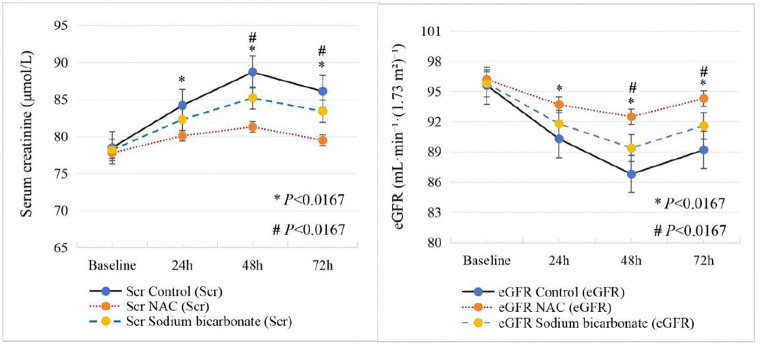
Dynamic changes in serum creatinine (Scr) and estimated glomerular filtration rate (eGFR) at baseline, 24 h, 48 h, and 72 h after contrast administration in the three groups. Data are presented as mean ± standard deviation. *P < 0.0167 (Bonferroni-corrected) compared with the control group at the same time point. **#**P < 0.0167 compared with the sodium bicarbonate group at the same time point. NAC = N-acetylcysteine.

1 Given the significant group × time interaction effects, simple effect analysis was performed with a Bonferroni correction. The dynamic changes in Scr and eGFR over time are illustrated in Fig 2.

2 **Intergroup simple effect**: At baseline, there were no significant differences in Scr, BUN, or eGFR across the three groups (all P > 0.0167). At 24 h, 48 h, and 72 h after contrast exposure:
  - The NAC group had significantly lower Scr and higher eGFR than the control group at all post-contrast time points (all P < 0.0167), with the most pronounced difference at 48 h and 72 h.
  - The sodium bicarbonate group had significantly lower Scr and higher eGFR than the control group at all post-contrast time points (all P < 0.0167);
  - The NAC group had significantly lower Scr and higher eGFR than the sodium bicarbonate group at 48 h and 72 h (all P < 0.0167), with no significant difference at 24 h (P > 0.0167);
  - Both the NAC and sodium bicarbonate groups had significantly lower BUN than the control group at all post-contrast time points (all P < 0.0167), with no significant difference between the two intervention groups at any time point (all P > 0.0167).

Intragroup simple effect: In all three groups, Scr and BUN levels at 24 h, 48 h, and 72 h after contrast were significantly higher than baseline (all P < 0.0167), while eGFR levels were significantly lower than baseline (all P < 0.0167). The absolute changes in Scr, BUN, and eGFR from baseline were largest in the control group and smallest in the NAC group, indicating the mildest renal function fluctuations in the NAC group.

### 3.3. Incidence of CA-AKI (Primary Outcome)

Per the 2024 ESUR diagnostic criteria [1], 12 patients in the control group developed CA-AKI (incidence 15.00%, 95% CI: 8.02%–24.78%), 2 patients in the NAC group (incidence 2.50%, 95% CI: 0.30%–8.74%), and 6 patients in the sodium bicarbonate group (incidence 7.50%, 95% CI: 2.79%–15.71%). The 15.00% CA-AKI incidence in the control group is consistent with the baseline data of Chinese low-risk patients with potential renal susceptibility factors, rather than the general low-risk population with no additional risk factors. [24].

Overall comparison revealed a significant difference in CA-AKI incidence across the three groups (Pearson’s χ²(2) = 8.235, P = 0.016). Post-hoc Bonferroni-corrected pairwise analyses (modified Poisson regression) showed:

- The NAC group had a significantly lower CA-AKI incidence than the control group (RR = 0.17, 95% CI: 0.04–0.74, adjusted P = 0.004 < 0.0167) [22];
- The sodium bicarbonate group had a numerically lower CA-AKI incidence than the control group, but the difference did not reach statistical significance after correction (RR = 0.50, 95% CI: 0.19–1.31, adjusted P = 0.121 > 0.0167) [16,36].
- There was no significant difference in CA-AKI incidence between the NAC and sodium bicarbonate groups after correction (adjusted P = 0.138 > 0.0167).

### 3.4. Subgroup analyses

#### 3.4.1. Subgroup analysis by underlying comorbidities.

Stratified analysis by diabetes and hypertension showed that in the non-diabetes and non-hypertension subgroups, the NAC group still had a significantly lower CA-AKI incidence than the control group (P < 0.0167). In the high-risk subgroups of patients with diabetes or hypertension [3,4], the NAC group had a numerically lower CA-AKI incidence than the other two groups, but the difference did not reach statistical significance after Bonferroni correction (all P > 0.0167) (Table 3). Multivariable logistic regression interaction term analysis showed no significant interaction effect between diabetes/hypertension status and treatment efficacy (all P for interaction > 0.05). Numerically, NAC was associated with a lower CA-AKI incidence in both the diabetes/hypertension subgroups and the non-diabetes/non-hypertension subgroups, though the statistical power for these subgroup analyses was 72% and 74%, respectively (below the pre-specified 80% threshold), limiting the ability to draw definitive conclusions about consistent efficacy across subgroups.

**Table 3:** CA-AKI incidence in subgroups stratified by underlying comorbidities [n (%)].

Subgroup	Group	Case number	CA-AKI cases (Incidence)	Corrected P value	P for interaction
Non-diabetes	Control group	60	7 (11.67)	0.120	0.321
	NAC Group	63	1 (1.59)		
	Sodium bicarbonate group	59	3 (5.08)		
Combined with diabetes	Control group	20	5 (25.00)	0.210	
	NAC Group	17	1 (5.88)		
	Sodium bicarbonate group	21	3 (14.29)		
Non-hypertension	Control group	50	6 (12.00)	0.113	0.415
	NAC Group	47	1 (2.13)		
	Sodium bicarbonate group	51	3 (5.88)		
Combined with hypertension	Control group	30	6 (20.00)	0.196	
	NAC Group	33	1 (3.03)		
	Sodium bicarbonate group	29	3 (10.34)		

Note: Bonferroni-corrected α’ = 0.0167; P < 0.0167 was considered statistically significant. P for interaction was calculated via the interaction term in the multivariable logistic regression model.

#### 3.4.2. Subgroup analysis by contrast modality.

Stratified analysis by contrast administration modality confirmed consistent protective efficacy of NAC across both routes [33]:

1. Contrast-enhanced CT subgroup (n = 132): CA-AKI incidence was 13.64% in the control group, 2.27% in the NAC group, and 6.82% in the sodium bicarbonate group, with a significant overall intergroup difference (Pearson’s χ²(2)=7.02, P = 0.031);
2. Angiography subgroup (n = 108): CA-AKI incidence was 16.67% in the control group, 2.78% in the NAC group, and 8.33% in the sodium bicarbonate group, with a significant overall intergroup difference (Pearson’s χ²(2)=7.15, P = 0.028).

The CA-AKI incidence was slightly higher in the angiography subgroup than the contrast-enhanced CT subgroup [33], consistent with the higher risk of intra-arterial contrast administration. Multivariable logistic regression interaction term analysis showed no significant interaction effect between contrast modality and treatment efficacy (P for interaction = 0.682), indicating the relative renoprotective effects of NAC and sodium bicarbonate were not affected by contrast administration modality.

### 3.5. Secondary clinical outcomes

During the 72-hour follow-up period, no patients in any group developed severe AKI (defined as KDIGO stage 3 AKI [26]) secondary to CA-AKI, and no patients required RRT. The mean hospital stay duration was 5.2 ± 1.3 days in the control group, 4.8 ± 1.1 days in the NAC group, and 5.0 ± 1.2 days in the sodium bicarbonate group. One-way ANOVA showed no significant difference in hospital stay duration across the three groups (F = 0.685, P = 0.505), indicating no significant impact of the three prophylaxis regimens on hospital stay in this mild-to-moderate risk population [7].

### 3.6. Independent risk factors for CA-AKI

Multivariable binary logistic regression analysis (VIF < 5 for all variables, no significant multicollinearity) revealed that advanced age (per 1-year increase, OR=1.025, 95% CI: 1.003–1.048, P = 0.004), comorbid diabetes (OR=1.892, 95% CI: 1.105–3.238, P = 0.012), comorbid hypertension (OR=1.765, 95% CI: 1.021–3.052, P = 0.028) [3,4], higher baseline Scr (per 1 μmol/L increase, OR=1.036, 95% CI: 1.012–1.061, P < 0.001), and lower baseline eGFR (per 1 mL·min ⁻ ¹·(1.73 m²)⁻¹ decrease, OR=0.968, 95% CI: 0.947–0.989, P < 0.001) [37] were independent risk factors for CA-AKI.

After adjusting for the above confounding factors, the protective effect of NAC against CA-AKI remained statistically significant (OR = 0.17, 95% CI: 0.04–0.74, P = 0.004), confirming that the renoprotective effect of NAC was independent of baseline risk factors. The protective effect of sodium bicarbonate did not reach statistical significance after adjustment (OR=0.50, 95% CI: 0.19–1.31, P = 0.121) (Table 4).

**Table 4:** Multivariable binary logistic regression analysis of independent risk factors for CA-AKI.

Variable	OR (95% CI)	P value	VIF
Intervention group (NAC vs Control)	0.17 (0.04–0.74)	0.004	1.05
Intervention group (Sodium bicarbonate vs Control)	0.50 (0.19–1.31)	0.121	1.04
Age (per 1-year increase)	1.025 (1.003–1.048)	0.004	1.12
Gender (Male vs. Female)	1.082 (0.513–2.283)	0.835	1.08
Diabetes (Yes vs No)	1.892 (1.105–3.238)	0.012	1.15
Hypertension (Yes vs No)	1.765 (1.021–3.052)	0.028	1.14
Coronary heart disease (Yes vs No)	1.153 (0.532–2.497)	0.716	1.09
Baseline Scr (per 1 μmol/L increase)	1.036 (1.012–1.061)	<0.001	1.21
Baseline eGFR (per 1 mL·min ⁻ ¹·(1.73 m²)⁻¹) decrease	0.968 (0.947–0.989)	<0.001	1.23
Contrast medium dosage (per 1 mL increase)	1.002 (0.991–1.013)	0.785	1.06
NYHA classification (Ⅱ vs Ⅰ)	1.215 (0.587–2.518)	0.602	1.07
ACEi/ARB use (Yes vs No)	1.108 (0.523–2.350)	0.789	1.08
Beta-blocker use (Yes vs No)	1.056 (0.498–2.240)	0.887	1.07

Note: OR = odds ratio; CI = confidence interval; ACEi/ARB = angiotensin-converting enzyme inhibitor/angiotensin receptor blocker; NYHA = New York Heart Association; VIF = variance inflation factor. VIF < 5 indicates no significant multicollinearity.

Notably, “lower baseline eGFR” in this study refers to eGFR in the lower normal range or mildly impaired (30–89 mL·min ⁻ ¹·(1.73 m² ⁻ ¹), as all patients with eGFR < 30 were excluded [24]. Contrast medium dosage, gender, coronary heart disease, NYHA classification, and baseline medication use (ACEi/ARB, beta blockers) were not independent risk factors for CA-AKI in this cohort (all P > 0.05).

### 3.7. Safety analysis: Adverse events

The overall incidence of adverse events was low across all groups, with no significant difference in the overall incidence among the three groups (Fisher’s exact test, P = 0.880). All adverse events were mild, with no moderate or severe events reported [22,30]. Specifically, 3 patients (3.75%) in the control group had adverse events (2 nausea, 1 headache), 2 patients (2.50%) in the NAC group (1 nausea, 1 rash), and 3 patients (3.75%) in the sodium bicarbonate group (2 nausea, 1 hypokalemia) [30]. All adverse events resolved spontaneously without specific treatment, except for the hypokalemia case in the sodium bicarbonate group, which resolved completely after oral potassium supplementation (Table 5).

**Table 5:** Incidence of adverse events in the three groups (n = 80 per group).

Type of adverse event	Control group [n (%)]	NAC group [n (%)]	Sodium bicarbonate group [n (%)]
Nausea	2 (2.50)	1 (1.25)	2 (2.50)
Headache	1 (1.25)	0 (0.00)	0 (0.00)
Rash	0 (0.00)	1 (1.25)	0 (0.00)
Hypokalemia	0 (0.00)	0 (0.00)	1 (1.25)
Total	3 (3.75)	2 (2.50)	3 (3.75)

## 4. Discussion

### 4.1. Principal findings

In this single-center retrospective study with 1:1:1 PSM adjustment for confounding, we found that hydration combined with high-dose intravenous NAC significantly reduced the 72-hour incidence of CA-AKI by 83% compared with conventional hydration alone in patients with eGFR ≥ 30 mL·min ⁻ ¹·(1.73 m²)⁻¹ [22]. Multivariable regression analysis confirmed that the renoprotective effect of NAC remained statistically significant after adjusting for baseline confounding factors, further verifying the robustness of this core finding. Hydration plus sodium bicarbonate showed a numerical 50% reduction in CA-AKI incidence and significantly milder renal function fluctuations, but the difference in the primary endpoint did not reach statistical significance after multiple testing correction [16,36]. Both regimens had a favorable safety profile, with only mild, self-limiting adverse events reported [22,30]. Numerically, the renoprotective effect of NAC was observed across both diabetes/hypertension subgroups and contrast administration modalities (intravenous CT vs. intra-arterial angiography), with no significant interaction effects detected between treatment and subgroup variables. However, the statistical power of diabetes/hypertension subgroup analyses was insufficient, so definitive conclusions about consistent efficacy across all subgroups cannot be made. We also identified advanced age, comorbid diabetes, comorbid hypertension, higher baseline Scr, and lower baseline eGFR as independent risk factors for CA-AKI in this mild-to-moderate risk cohort [3,4,37], which is consistent with the results of international and Chinese population studies [24].

### 4.2. Renoprotective efficacy of NAC: Mechanisms and clinical context

The significant renoprotective effect of high-dose intravenous NAC observed in this study aligns with recent meta-analyses confirming that high-dose intravenous NAC is superior to low-dose oral NAC for CA-AKI prevention in high-risk patients [20,21]. The superiority of this regimen is attributed to three key factors: first, intravenous administration avoids the first-pass effect of oral dosing [21], achieving rapid, stable, and high plasma concentrations to ensure sufficient sulfhydryl groups for ROS scavenging and glutathione synthesis [12]; second, the 12 g dose provides sufficient antioxidant capacity to target the core oxidative stress pathogenesis of CA-AKI [9,10], which is not fully addressed by hydration alone; third, NAC improves renal medullary perfusion via nitric oxide-mediated vasodilation [13], mitigating the ischemia-hypoxia injury induced by contrast media [8]. In addition, the timing of NAC administration (1 hour before contrast exposure) in this study is also an important factor for its good efficacy [32], as pre-administration ensures that the drug reaches effective plasma concentrations before contrast exposure.

Notably, our findings differ from the landmark PRESERVE trial [17], which found no significant renoprotective effect of NAC or sodium bicarbonate on 90-day severe renal adverse events. A reanalysis of the PRESERVE trial further explained this discrepancy [18], which is also due to critical differences in study design: first, the PRESERVE trial focused on long-term (90-day) severe renal outcomes [17], while our study targeted the short-term (72-hour) CA-AKI incidence [1], which is the direct, immediate endpoint of contrast-induced renal injury per international guidelines; second, the PRESERVE trial enrolled severe high-risk patients with eGFR < 60 mL·min ⁻ ¹·(1.73 m²)⁻¹ and comorbid diabetes/heart failure [17], while our study included mild-to-moderate risk patients with eGFR ≥ 30 mL·min ⁻ ¹·(1.73 m²)⁻¹ [24], where NAC may have more pronounced renoprotective effects; third, the PRESERVE trial used low-dose oral NAC (600 mg twice daily) [17], while our study used high-dose intravenous NAC [22], which has consistently shown superior efficacy in prior meta-analyses [20,21].

### 4.3. Renoprotective efficacy of sodium bicarbonate: A viable alternative

Our study found that hydration plus sodium bicarbonate resulted in a numerical reduction in CA-AKI incidence and significantly milder renal function fluctuations compared with hydration alone, but the difference in the primary endpoint did not reach statistical significance after Bonferroni correction [16,36]. This is consistent with recent meta-analyses showing inconsistent prophylactic efficacy of sodium bicarbonate across studies [38].

The partial renoprotective effect of sodium bicarbonate is attributed to its urinary alkalinization effect [1,14], which reduces contrast media crystallization and tubular deposition and indirectly inhibits ROS production in an alkaline environment [15,16]. However, its efficacy is numerically inferior to NAC, likely because it only targets one component of CA-AKI pathogenesis (crystallization and intratubular obstruction) [11], while NAC addresses the core mechanisms of oxidative stress [9,10,12] and medullary ischemia-hypoxia [8,13]. In addition, the efficacy of sodium bicarbonate is highly dependent on the maintenance of the target urine pH, which may be affected by individual differences in renal tubular function, leading to inconsistent efficacy across patients.

For patients intolerant to NAC (e.g., with a history of allergic reactions or severe gastrointestinal adverse events), sodium bicarbonate remains a safe and viable alternative [1,14]. However, strict monitoring of urine pH and serum electrolyte levels is recommended during treatment, as sodium bicarbonate may cause hypokalemia and hypernatremia [30], especially in patients with mild renal impairment. This is consistent with the 2024 ESUR guidelines [1,14], which recommend sodium bicarbonate as a second-line prophylaxis option for patients with NAC contraindications.

### 4.4. Interpretation of the 15% CA-AKI incidence in the control Group

The 15.00% CA-AKI incidence in the hydration alone control group is higher than the 1%–3% incidence reported in the general low-risk population with no additional renal susceptibility factors [4], but is fully explained by four key factors and is consistent with the baseline incidence of Chinese low-risk patients with potential susceptibility factors [24]:

1. Latent renal susceptibility factors: Although free of diabetes/hypertension, 68.75% of control group patients were aged ≥50 years, and 18.75% had underlying cardiovascular disease, which are known to increase renal vulnerability to contrast media [39,35].
2. High-contrast medium dosage at the upper limit of the group definition: The median contrast dosage in the control group was 92.5 mL (IQR: 85.0–98.0 mL), close to the 100 mL threshold, and a dose-response meta-analysis confirmed that doses >90 mL are associated with increased CA-AKI risk in low-risk populations [23].
3. Fixed-rate hydration without individualization;
4. Ethnic population characteristics: Chinese low-risk populations have a relatively higher baseline CA-AKI incidence compared with Western populations, as reported in previous cohort studies [24].

### 4.5. Clinical implications

Our findings have important clinical implications for CA-AKI prophylaxis in mild-to-moderate-risk patients with eGFR ≥ 30 mL·min ⁻ ¹·(1.73 m²)⁻¹, especially for Chinese populations [24]:

1. For patients with comorbid diabetes/hypertension or those receiving high-dose contrast media (≥1.5 mL/kg) [22], hydration combined with high-dose intravenous NAC should be considered as a first-line prophylactic regimen [23], given its significant renoprotective effect and favorable safety profile [32].
2. For patients intolerant to NAC, hydration combined with sodium bicarbonate is a safe alternative [1,14], with strict monitoring of urine pH and serum electrolytes during treatment [30].
3. Conventional hydration alone may be insufficient for latent high-risk patients with normal renal function but comorbid diabetes/hypertension [39], and individualized hydration optimization [31] or combined nephroprotective agents should be considered [6,7].
4. The protective efficacy of NAC is consistent across both contrast-enhanced CT and angiography [33], eliminating the need to adjust the prophylactic regimen based on the contrast administration modality.
5. Notably, the three prophylactic regimens showed no significant difference in hospital stay duration (P = 0.505), indicating that the application of NAC or sodium bicarbonate on the basis of hydration will not increase the hospital stay of mild-to-moderate-risk patients.

### 4.6. Study limitations

This study has several important limitations that must be acknowledged:

1. Single-center retrospective design: Despite the use of PSM to minimize confounding bias, selection bias, and residual confounding from unmeasured variables are inevitable due to the non-randomized grouping, which may limit the external generalizability of our findings to other clinical settings [40]. In addition, group allocation was based on institutional clinical protocol rather than randomization, even after PSM adjustment for measured baseline confounders, residual confounding from unmeasured variables (such as patients’ baseline volume status, contrast injection rate, and perioperative blood pressure fluctuations) may still exist. However, we minimized this risk by: (1) using protocol-based grouping to reduce physician bias; (2) including all known major CA-AKI risk factors in the PSM model; (3) achieving excellent balance of all measured covariates after matching (all SMD < 0.1).
2. Limited sample size and CA-AKI events: The sample size was 80 patients per group, with a total of only 20 CA-AKI events across all groups, which may have led to insufficient statistical power for some subgroup analyses. Post-hoc power analysis confirmed a statistical power of 72% for the diabetic subgroup and 74% for the hypertensive subgroup, both below the pre-specified 80% threshold, which may have led to type II error and false-negative results in these high-risk subgroup analyses.
3. Short follow-up duration: The study only followed patients for 72 hours to assess the primary CA-AKI endpoint [1,26], with no long-term follow-up data to evaluate the impact of the different regimens on long-term renal outcomes (CKD progression, RRT requirement, and all-cause mortality) [2,41].
4. Restricted study population: Patients with severe renal dysfunction (eGFR < 30 mL·min ⁻ ¹·(1.73 m² ⁻ ¹)) [28,29] and NYHA class Ⅲ–Ⅳ cardiac function were excluded, so the results cannot be directly extrapolated to these severe high-risk populations [3].
5. Incomplete AKI assessment: The urine output criterion (urine output <0.5 mL/kg/h for 48 hours) required by the 2024 ESUR guidelines for CA-AKI diagnosis was not applied due to the lack of complete timed urine collection data in the retrospective electronic medical record system (EMRS). CA-AKI was only diagnosed based on serum creatinine changes (a relative increase of ≥25% or an absolute increase of ≥44.2 μmol/L within 72 hours), which may lead to an underestimation of the true incidence of CA-AKI and results in an incomplete implementation of the 2024 ESUR diagnostic criteria [1,26];
6. Lack of novel AKI biomarkers: The study did not measure early AKI biomarkers such as urinary NGAL or KIM-1 [34], which provide more sensitive and earlier assessment of contrast-induced renal injury [34,42];
7. No cost-effectiveness analysis: The study did not evaluate the cost-effectiveness of the three prophylactic regimens [43], which is an important factor for clinical routine application.
8. The statistical power for subgroup analyses of patients with diabetes and hypertension was 72% and 74%, respectively, below the pre-specified 80% threshold. This may have led to a type II error, and thus the numerical trends of NAC efficacy in these high-risk subgroups need to be verified in larger-sample studies.

### 4.7. Future research directions

Based on the limitations of this study and the latest research progress [44,45], future research should focus on the following areas:

1. Large-sample, multicenter, prospective RCTs to verify the renoprotective efficacy of high-dose intravenous NAC and sodium bicarbonate across different risk stratifications, especially in severe high-risk patients with eGFR < 60 mL·min ⁻ ¹·(1.73 m²)⁻¹ [18,44];
2. Long-term follow-up studies (6–12 months) to evaluate the impact of different prophylaxis regimens on long-term renal outcomes, including CKD progression, RRT requirement, and all-cause mortality [4,41,46];
3. Subgroup analyses with sufficient sample size to identify patient populations that derive the greatest benefit from NAC or sodium bicarbonate [5,6] to inform personalized clinical recommendations [40];
4. Studies incorporating novel AKI biomarkers to explore the early predictive value of these markers for CA-AKI [34,42], and to further elucidate the mechanisms of action of NAC and sodium bicarbonate [9,47];
5. Cost-effectiveness analyses of different CA-AKI prophylaxis regimens to inform health economic decision-making [43];
6. Research on the optimal administration timing and dosage of NAC to further improve its renoprotective efficacy [32] and explore the combination of NAC with other nephroprotective agents [45].

## 5. Conclusions

In conclusion, this single-center retrospective study with PSM adjustment for confounding demonstrates that for patients with eGFR ≥ 30 mL·min ⁻ ¹·(1.73 m²)⁻¹, undergoing contrast-enhanced CT or angiography [24], hydration combined with high-dose intravenous NAC significantly reduces the short-term incidence of CA-AKI compared with conventional hydration alone [22,32], with a favorable safety profile and consistent efficacy across different contrast modalities [33]. Multivariable regression analysis confirmed that the renoprotective effect of NAC is independent of baseline risk factors, further verifying the robustness of this finding. Hydration combined with sodium bicarbonate shows a numerical reduction in CA-AKI incidence and is a safe alternative for patients intolerant to NAC [1,14,36], with strict monitoring of urine pH and electrolyte levels recommended during treatment [30]. Advanced age, comorbid diabetes, comorbid hypertension, higher baseline Scr, and lower baseline eGFR are independent risk factors for CA-AKI in this mild-to-moderate risk population [3,4,37]. Due to the single-center retrospective design, these findings are hypothesis-generating and require further verification in large-sample, multicenter, prospective RCTs with long-term follow-up [44,45].

## Supporting information

S1 File Raw Data. (XLSX)

## Abbreviations

CA-AKI: Contrast-associated acute kidney injury (historically termed contrast-induced nephropathy, CIN)
NAC: N-Acetylcysteine
GFR: Estimated glomerular filtration rate
PSM: Propensity score matching
Scr: Serum creatinine
BUN: Blood urea nitrogen
KDIGO: Kidney Disease: Improving Global Outcomes
ESUR: European Society of Urogenital Radiology
ROS: Reactive oxygen species
CKD: Chronic kidney disease
RRT: Renal replacement therapy
NYHA: New York Heart Association
LVEF: Left ventricular ejection fraction
ACEi/ARB: Angiotensin-converting enzyme inhibitor/angiotensin receptor blocker
SMD: Standardized mean difference
ULN: Upper limit of normal
ANOVA: Analysis of variance
CI: Confidence interval
OR: Odds ratio
RR: Relative risk
STROBE: Strengthening the Reporting of Observational Studies in Epidemiology
IQR: Interquartile Range
VIF: Variance Inflation Factor
CONSORT: Consolidated Standards of Reporting Trials
EMR: Electronic Medical Record System
