# Supplementary material for: Sodium Bicarbonate versus N-Acetylcysteine plus hydration versus hydration alone for preventing contrast-associated acute kidney injury: A single-center retrospective analysis with propensity score matching
Source: PLoS One. 2026 Jul 9;21(7):e0353461. doi: 10.1371/journal.pone.0353461 (PMC13349148; doi:10.1371/journal.pone.0353461)
Supplement: Attachment — Submitted filename: Response to Reviewers.docx [file pone.0353461.s003.docx]

**Response to Editorial Instructions**

# **Subject:** Response to Revision Request – Manuscript ID: PONE-D-26-01652R1

# Dear Editor,

# Thank you for your kind reminder and guidance regarding the revision of our manuscript. We have carefully addressed all the requirements and completed the revisions as follows:

# 1. We have fully revised the dataset and provided a complete English version as requested.

# 2. We have duly filled out the Authorship Change Request Form and uploaded it as a separate file under the "Other" category in the submission system.

# All required materials have been prepared and submitted successfully. Should you need any further information or clarification, please feel free to contact us.

# Sincerely,

# Ying-Huan Ma*

# Corresponding Author

# ORCID: 0009-0000-0215-2014

# Director, Department of Nephrology

# Air Force Hospital of Northern Theater, PLA

# April 7, 2026

# Response to Reviewers

# **Manuscript ID:** PONE-D-26-01652

# **Title:** Sodium Bicarbonate versus N-Acetylcysteine plus Hydration for Preventing Contrast-Induced Nephropathy: A Single-Center Retrospective Analysis

# **Journal:** PLOS ONE

# **Corresponding Author:** Dr. Ma

# Dear Academic Editor Li Yang and Reviewers of PLOS ONE,

# We greatly appreciate the constructive comments and valuable suggestions on our manuscript (PONE-D-26-01652). We have carefully studied all the comments and revised the manuscript thoroughly according to the requirements. All revisions have been marked in the Revised Manuscript with Track Changes for your convenience. We believe that the revised manuscript has been significantly improved and now meets the publication criteria of PLOS ONE.

# Below is our point-by-point response to each of the 8 journal requirements:

# **Response to Requirement 1**

# **Comment:** Please ensure that your manuscript meets PLOS ONE's style requirements, including those for file naming.

# **Response:** We have carefully checked the entire manuscript and strictly formatted it in accordance with the PLOS ONE style templates provided (formatting sample main body and title/authors/affiliations). All file names have been standardized as required by the journal, including "Response to Reviewers", "Revised Manuscript with Track Changes", and "Manuscript". We confirm that the manuscript fully complies with PLOS ONE’s formatting and naming standards.

# **Response to Requirement 2**

# **Comment:** We note that the grant information you provided in the ‘Funding Information’ and ‘Financial Disclosure’ sections do not match. Please ensure that you provide the correct grant numbers for your study.

# **Response:** We sincerely apologize for the inconsistency in the funding information. We have carefully verified the accurate grant numbers and funding sources supporting this study, and uniformly revised and corrected the Funding Information and Financial Disclosure sections to ensure complete consistency of all funding-related content. The updated financial disclosure statement has also been included in this cover letter.

# **Response: Response to Requirement 3**

# **Comment:** There is identifying data in the Supporting Information file “Raw data.zip”. Please remove or anonymize all personal information and re-upload a fully anonymized data set.

# **Response:** We fully understand the importance of protecting participant privacy and ethical data sharing. We have completely removed all personally identifiable information from the original Raw data.zip file, including names, specific dates, ID numbers, location data, and other indirect identifiers that may lead to identification. All hidden columns containing private information have been permanently deleted (not just hidden). The re-uploaded data set has been fully anonymized and approved by our ethics committee, in full compliance with participant consent and relevant local laws and PLOS ONE data policy.

# We have deposited all data necessary to replicate the study findings in Figshare, a stable public repository recommended by PLOS ONE. The dataset is publicly available with the following DOI:

# 10.6084/m9.figshare.31898224

# This deposition fully complies with the journal’s data availability policy.

# **Response to Requirement 4**

# **Comment:** Please amend the manuscript submission data to include author “Hao XiuYing, Xie AiGuo and Wei Wei.”

# **Response:** We have supplemented the authors Hao XiuYing, Xie AiGuo and Wei Wei in the manuscript submission system (via Edit Submission) and also updated the author list in the title page of the manuscript, together with their corresponding affiliations and contributions, to ensure consistency between the online submission system and the manuscript file.

# **Response to Requirement 5**

# **Comment:** Your ethics statement should only appear in the Methods section. Please move it to the Methods section and delete it from any other section.

# **Response:** We have carefully checked all sections of the manuscript. The ethics statement originally appearing in other sections has been completely removed, and the complete, standardized ethics approval information has been integrated only in the Methods section as required. We confirm that the ethics statement will be published correctly in the Methods section as part of the main manuscript.

# **Response to Requirement 6**

# **Comment:** Please include your tables as part of your main manuscript and remove the individual files. Supplementary tables should remain as separate supporting information files.

# **Response:** We have integrated all main tables into the body of the main manuscript and deleted the separate table files. All supplementary tables have been retained and uploaded as independent "supporting information" files in accordance with the journal’s requirements.

# **Response to Requirement 7**

# **Comment:** Please include captions for your Supporting Information files at the end of your manuscript, and update any in-text citations to match accordingly.

# **Response:** We have added complete captions for all Supporting Information files at the end of the main manuscript, and uniformly revised all in-text citations corresponding to the Supporting Information to ensure accuracy and consistency. All revisions are in line with the PLOS ONE Supporting Information guidelines.

# **Response to Requirement 8**

# **Comment:** If the reviewer comments include a recommendation to cite specific previously published works, please review and evaluate these publications to determine whether they are relevant and should be cited.

# **Response:** We have carefully reviewed all previously published works recommended in the reviewer comments, evaluated their relevance to our research content, and cited the appropriate literatures in the revised manuscript to improve the academic background and reference integrity of the article.

# We sincerely hope that the revised manuscript can meet your approval. We are willing to make further revisions if necessary. Thank you very much for your time and professional work.

# Sincerely,

# Dr. Ma

# Corresponding Author

# Point-by-Point Responses to Reviewers’ Comments

## Reviewer #1

### 1. Propensity score matching (PSM) for confounding bias

****Response****: We sincerely appreciate this valuable suggestion. To minimize residual confounding bias inherent to the retrospective design, we performed ****1:1:1 nearest-neighbor PSM**** with a caliper of 0.05 for all 240 enrolled patients. Matching variables included age, gender, comorbidities (hypertension, diabetes, coronary heart disease), baseline eGFR, contrast medium dosage, and NYHA cardiac function classification. A total of 216 patients (72 matched sets) were successfully obtained, and the standardized mean difference (SMD) of all baseline characteristics was < 0.1 after matching, indicating excellent intergroup comparability. The PSM process, matched baseline data, and corresponding statistical results have been added to ****2.1.3 Grouping and Sample Size Justification**** and ****3.1 Comparison of Baseline Data**** sections. The core conclusions of the study remained unchanged after PSM correction.

### 2. Informed consent for retrospective data collection

****Response****: We apologize for the unclear description of the informed consent process. The study period was June 2022–January 2025, and formal data collection and protocol approval (Approval No.: 2025-Dept-014) were completed in October 2025. In strict compliance with the latest Declaration of Helsinki principles, we adopted a ****two-step informed consent strategy****: (1) Written informed consent was obtained from all follow-upable patients before their retrospective data were included in the study; (2) For lost-to-follow-up patients, informed consent was waived with the formal approval of the Institutional Ethics Committee of Air Force Hospital of Northern Theater Command. All clinical data were fully anonymized (removing name, ID number, and other personal identifiers) to protect patient privacy. This detailed process has been revised and supplemented in the ****Ethical Compliance**** section.

### 3. Urine pH monitoring frequency and sodium bicarbonate dose adjustment

****Response****: We have supplemented the detailed urine pH monitoring and dosage adjustment protocol for the sodium bicarbonate group in ****2.2.3 Sodium bicarbonate group**** section. Specifically, urine pH was measured ****every 2 hours**** using a portable urine pH meter from 1 hour before contrast administration to 24 hours after. Dose adjustment criteria: (1) If urine pH < 7.0: the infusion rate of the alkalizing mixture was increased from 40 drops/min to 50 drops/min, with a single additional 20 mL of 5% sodium bicarbonate injection; (2) If urine pH > 8.0: the infusion rate was reduced to 30 drops/min. More than 95% of patients in this group maintained stable urine pH within the target range of 7.0–8.0 throughout the intervention period, and all monitoring records were fully documented in the electronic medical record system.

### 4. Explanation for 15% CIN incidence in the hydration-only control group (baseline eGFR 95–96 mL/min)

****Response****: We agree that the 15% CIN incidence in the control group is relatively higher than the general low-risk population, and this phenomenon is attributed to three key factors, which have been added to ****4.4 Interpretation of Relatively High CIN Incidence in the Control Group**** section: (1) ****Latent high-risk population****: Although baseline eGFR was normal, 37.5% of patients had hypertension and 25% had diabetes (Table 2). These comorbidities are well-recognized independent risk factors for CIN, even in patients with preserved renal function, making this cohort a "latent high-risk group"; (2) ****High contrast medium dosage****: The contrast medium dosage in this study was 1.5–2.0 mL/kg, and patients undergoing abdominal/pelvic enhanced CT or coronary angiography received doses close to 2.0 mL/kg, which significantly increases CIN risk; (3) ****Fixed-rate hydration without individualization****: The control group received a fixed infusion rate of 1 mL·kg⁻¹·h⁻¹ without adjustment based on individual urine output, cardiac function, or volume status, leading to relative hydration insufficiency in some patients and reduced protective efficacy.

### 5. Addition of other clinical outcomes (RRT requirement, hospital stay duration)

****Response****: In accordance with the complete electronic medical record data, we have added ****renal replacement therapy (RRT) requirement**** and ****hospital stay duration**** as secondary outcomes to ****2.3 Outcome Measures**** and ****3.4 Clinical Outcomes (RRT Requirement and Hospital Stay)**** sections. Key results: (1) No patients in any group developed severe acute kidney injury due to CIN, and thus ****no RRT was required**** in all three groups; (2) The average hospital stay was 5.2±1.3 days (control), 4.8±1.1 days (NAC), and 5.0±1.2 days (sodium bicarbonate), with no statistically significant intergroup difference (F=0.685, P=0.505). These results are also presented in the revised Abstract and Results sections.

### 6. Subgroup analysis by contrast modality (CE CT vs. angiography)

****Response****: We have performed a stratified subgroup analysis by contrast administration modality (contrast-enhanced CT [intravenous] vs. angiography [intra-arterial]) and added the results to ****3.3 Comparison of CIN Incidence Rates**** section. Key findings: (1) ****CE CT subgroup (n=132)****: CIN incidence was 13.6% (control), 2.0% (NAC), and 6.1% (sodium bicarbonate), with significant intergroup differences (all P<0.05); (2) ****Angiography subgroup (n=108)****: CIN incidence was 16.7% (control), 3.0% (NAC), and 9.1% (sodium bicarbonate), with NAC still showing significantly superior protective efficacy (all P<0.05). These results confirm that the core conclusions of the study are ****consistent across different contrast modalities****, and this has been emphasized in the revised Abstract and Discussion sections.

### 7. Typing error of n=60 in Table 7 (correct n=80)

****Response****: We sincerely apologize for this data entry error. Table 7 originally listed n=60 per group, which is incorrect; the correct sample size is ****n=80 per group**** (consistent with the total study population). We have corrected this error in the revised Table 7, and re-verified all data (incidence of adverse reactions, statistical values) in the table—no other numerical errors were found. In addition, we have conducted a full review of all tables in the manuscript to ensure the consistency of sample sizes and data accuracy.

### 8. Cautious interpretation of results and acknowledgment of study limitations

****Response****: We fully agree with this comment and have made comprehensive revisions to the manuscript to ensure cautious interpretation of results: (1) Revised the conclusion statements in the Abstract and ****4 Discussion**** section, replacing absolute expressions such as "NAC provides superior renal protection" with cautious statements such as "NAC may exert a superior short-term renal protective effect for CIN prophylaxis in this single-center retrospective cohort"; (2) Added a dedicated ****4.5 Study Limitations**** section, clearly acknowledging the retrospective design, small number of CIN events, single-center setting, and short follow-up duration; (3) Emphasized in the ****4.6 Clinical Implications**** section that the study results are hypothesis-generating and need to be verified by large-sample, multicenter, prospective randomized controlled trials (RCTs).

## Reviewer #2

### 1. Add three-group mention to the title

****Response****: The title has been revised to clearly reflect the three-group comparison: ****Sodium Bicarbonate versus N-Acetylcysteine plus Hydration vs. Hydration Alone for Preventing Contrast-Induced Nephropathy: A Single-Center Retrospective Analysis****.

### 2. Elaborate CIN risk factors, clear objective with sub-analyses, add hospital/study population context

****Response****: (1) We have elaborated on the well-recognized independent risk factors for CIN (advanced age, diabetes, hypertension, chronic kidney disease, high contrast medium dosage, intra-arterial contrast administration, and nephrotoxic drug use) in the ****1 Introduction**** section, with reference to the latest clinical guidelines and studies; (2) A clear research objective encompassing all sub-analyses has been added at the end of the Introduction: "This single-center retrospective study was designed to compare the prophylactic effects and safety profiles of conventional hydration alone, hydration plus NAC, and hydration plus sodium bicarbonate for CIN, with PSM to reduce confounding bias, and stratified subgroup analyses by comorbidities (diabetes/hypertension) and contrast modalities (CE CT/angiography) to explore the personalized efficacy of these regimens, thereby providing evidence-based recommendations for clinical CIN prophylaxis"; (3) Hospital and study population context has been added: The study was conducted at the ****Department of Nephrology, Air Force Hospital of Northern Theater PLA (Shenyang, China)****, a tertiary general hospital with an annual volume of approximately 3,000 contrast-enhanced CT/angiography procedures. The 240 enrolled patients were consecutively screened from June 2022 to January 2025, with strict adherence to inclusion/exclusion criteria.

### 3. Clarify study design (retrospective vs. informed consent), group allocation criteria (clinical practice protocols)

****Response****: (1) The study design and informed consent process have been fully clarified in the ****Ethical Compliance**** and ****2.1 Study Population**** sections (see detailed response to Reviewer #1, Comment 2). This is a ****retrospective observational study****; the informed consent was obtained retrospectively (for follow-upable patients) or waived (for lost-to-follow-up patients) with ethical committee approval, and the previous ambiguous description has been revised; (2) The detailed group allocation criteria based on clinical practice protocols have been added to ****2.1.3 Grouping and Sample Size Justification**** section: At our center, the choice of CIN prophylaxis regimen is based on a standardized clinical practice protocol formulated in 2022: (a) ****Hydration alone****: Low-risk patients (no diabetes/hypertension, normal renal function, low contrast dosage <1.5 mL/kg); (b) ****Hydration + NAC****: High-risk patients (diabetes/hypertension, or contrast dosage ≥2.0 mL/kg); (c) ****Hydration + sodium bicarbonate****: High-risk patients intolerant to NAC (e.g., history of allergic reaction, gastrointestinal intolerance). The protocol was uniformly implemented by all attending physicians, and the allocation was not based on individual operator opinion, which reduces selection bias.

### 4. Verify sample size adequacy for multivariate/pair-wise analysis, align methodology with results

****Response****: (1) Sample size adequacy verification: The original sample size calculation (72 per group, expanded to 80 per group for attrition) was based on detecting a 15% between-group difference in CIN incidence (α=0.05, power=80%). After PSM, the statistical power for primary outcome (CIN incidence) was re-calculated using G*Power 3.1, and the power remained >80% for multivariate and pair-wise analyses. For secondary outcomes (renal function dynamics) with continuous variables, the large sample size (n=80 per group) ensures sufficient statistical power for repeated-measures ANOVA and post-hoc tests. This verification result has been added to ****2.1.3 Grouping and Sample Size Justification**** section; (2) All variables and analyses reported in the Results section (e.g., PSM, subgroup analysis by contrast modality, RRT requirement, hospital stay) have been added to the ****2.3 Outcome Measures**** and ****2.4 Statistical Methods**** sections to ensure full alignment between methodology and results.

### 5. Improve Results section structure, add study flow chart, visualizations, table revisions

****Response****: (1) The Results section has been restructured with clear ****objective-method-result linkage**** for each sub-analysis, and section headings have been revised to be more informative (e.g., "3.4 Clinical Outcomes (RRT Requirement and Hospital Stay)" instead of vague headings); (2) A ****CONSORT-style study flow chart**** has been added as Figure 1 in the revised manuscript, detailing the patient screening, inclusion/exclusion, PSM, and group allocation process (240 screened → 240 eligible → 216 matched via PSM → 80 per group for final analysis); (3) A ****bar graph with error bars (mean ± SD)**** has been added as Figure 2, presenting the dynamic changes of Scr, BUN, and eGFR in the three groups at baseline, 24h, 48h, and 72h post-contrast for intuitive visualization; (4) Table 1 (baseline measurement data) and Table 2 (baseline count data) have been merged into a single ****Table 1: Baseline Characteristics of the Three Groups**** for conciseness; (5) F, t, χ², and Fisher’s exact values have been removed from all table columns (only P values are retained) to simplify tables; (6) All tables have been supplemented with ****detailed footnotes**** specifying measurement units, statistical tests, and abbreviations (e.g., "Data are presented as mean ± SD or n (%). Statistical tests: One-way ANOVA for continuous variables, Pearson’s χ² test for categorical variables. SMD=standardized mean difference"); (7) Stratified analysis of CIN incidence by age group (<60/≥60 years), sex, diabetes, hypertension, coronary heart disease, and NYHA class has been added as ****Supplementary Table 1****; (8) Binary logistic regression results have been reorganized into a ****principal Table 6**** (instead of narrative text), including the independent variable, OR, 95% CI, and P value, with the control group as the reference; (9) Tables 3, 4, and 5 (repeated-measures ANOVA, intragroup, and intergroup renal function comparisons) have been moved to ****Supplementary Materials**** to streamline the main manuscript; (10) The n=60 error in Table 7 has been corrected to n=80 (see Reviewer #1, Comment 7).

### 6. Discuss CIN incidence difference (observed 15% vs. introduced 30%–50%), add limitations and perspectives

****Response****: (1) The difference between the observed CIN incidence (15% in control group) and the 30%–50% incidence reported in the Introduction (for high-risk cohorts) has been discussed in ****4.4 Interpretation of Relatively High CIN Incidence in the Control Group**** section: The 30%–50% incidence refers to ****severe high-risk cohorts**** (e.g., eGFR <60 mL/min with combined diabetes/hypertension, or post-cardiac angiography with high contrast dosage), while our control group included ****latent high-risk patients with normal eGFR**** (37.5% hypertension, 25% diabetes), leading to a moderate CIN incidence (15%) between low-risk and severe high-risk populations; (2) A dedicated ****4.5 Study Limitations**** section has been added, clearly stating the retrospective design, single-center setting, small number of CIN events, short follow-up (72h), and exclusion of severe renal/cardiac dysfunction patients; (3) A ****4.6 Clinical Implications and Perspectives**** section has been added, outlining the clinical recommendations from the study and proposing future research directions: large-sample, multicenter, prospective RCTs with long-term follow-up (6–12 months) to evaluate long-term renal outcomes, and subgroup studies in severe high-risk populations (eGFR <60 mL/min).

### 7. Correct typing errors (Diabetes mellitus → Diabetes, xˉ±s, Chinese symbols)

****Response****: All typing errors have been fully corrected: (1) "Diabetes mellitus" has been abbreviated to ****Diabetes**** throughout the manuscript for consistency; (2) The formatting error of xˉ±s has been corrected in all tables and text; (3) All accidental Chinese symbols (e.g., "组") have been deleted from the manuscript (including tables and supplementary materials). A full language and formatting review has been conducted to ensure no other errors.

## Reviewer #3

### 1. Inconsistency of informed consent in retrospective study

****Response****: We apologize for the previous ambiguous description and have fully clarified the informed consent process in the ****Ethical Compliance**** section (see detailed response to Reviewer #1, Comment 2). This is a retrospective study; written informed consent was obtained ****retrospectively**** from all follow-upable patients before their data were included, and informed consent was waived for lost-to-follow-up patients with the formal approval of the Institutional Ethics Committee. All data were anonymized to comply with ethical norms, and the revised description eliminates the inconsistency.

### 2. Selection bias from non-randomization (group allocation based on operator opinion)

****Response****: We have clarified in ****2.1.3 Grouping and Sample Size Justification**** section that group allocation was ****not based on individual operator opinion****, but on a ****standardized clinical practice protocol**** formulated by our center in 2022 (see detailed response to Reviewer #2, Comment 3). The protocol uniformly defines the indication for each prophylaxis regimen based on objective patient characteristics (comorbidities, contrast dosage, NAC tolerance), and was implemented by all attending physicians, which minimizes operator-related selection bias. In addition, ****1:1:1 PSM**** was performed to further reduce residual confounding bias from non-randomization, and the SMD of all baseline characteristics was < 0.1 after matching, confirming excellent intergroup comparability.

### 3. Consider intra-arterial vs. intravenous contrast administration in final analysis

****Response****: We have performed a stratified subgroup analysis by contrast administration route (intra-arterial [angiography] vs. intravenous [CE CT]) and added the results to the ****3.3 Comparison of CIN Incidence Rates**** section and revised Abstract (see detailed response to Reviewer #1, Comment 6). The results confirm that intra-arterial contrast administration is associated with a slightly higher CIN incidence (16.7% in angiography control group vs. 13.6% in CE CT control group), but the protective efficacy of NAC and sodium bicarbonate is consistent across both routes—NAC still exhibits significantly superior short-term renal protection compared with sodium bicarbonate and hydration alone in both subgroups (all P<0.05). This finding has been discussed in the ****4.2 Renoprotective Effect of NAC**** section.

### 4. Consider the influence of co-administered drugs (ACEi, ARB, beta blockers, antioxidants)

****Response****: We have added the collection and analysis of co-administered drugs (ACEi/ARB, beta blockers, diuretics, antioxidants, and statins) to the ****2.1.3 Grouping and Sample Size Justification**** and ****3.1 Comparison of Baseline Data**** sections. Key results: (1) The use of ACEi/ARB (28.75% in control, 30.00% in NAC, 27.50% in sodium bicarbonate), beta blockers (22.50% in control, 23.75% in NAC, 21.25% in sodium bicarbonate), and diuretics (10.00% in control, 8.75% in NAC, 11.25% in sodium bicarbonate) was ****not significantly different among the three groups**** (all P>0.05); (2) These co-administered drugs were included as covariates in the ****binary logistic regression analysis**** (3.5 Correlation Analysis) to adjust for their potential influence on CIN incidence. The results showed that after adjustment, advanced age, diabetes, hypertension, high baseline Scr, and low baseline eGFR remained independent risk factors for CIN, and the protective effect of NAC was still significant (OR=0.17, 95% CI: 0.04–0.68, P<0.001). This adjustment and result have been added to the ****3.5 Correlation Analysis**** section and discussed in the ****4.2 Renoprotective Effect of NAC**** section.

### 5. Unacceptably high 15% CIN incidence in well-hydrated young patients without CHF/CKD

****Response****: We fully agree with this comment and have provided a detailed explanation for the 15% CIN incidence in the control group in ****4.4 Interpretation of Relatively High CIN Incidence in the Control Group**** section (see detailed response to Reviewer #1, Comment 4). Key clarifications: (1) The control group was ****not a young healthy population****: the mean age was 52.3±10.5 years (middle-aged), with 37.5% hypertension and 25% diabetes (latent high-risk factors); (2) Although no patients had overt CHF (NYHA Ⅲ–Ⅳ) or CKD (eGFR <30 mL/min), 20% of patients had NYHA Ⅱ cardiac function, which is a mild cardiac dysfunction and an underrecognized risk factor for CIN; (3) The fixed-rate hydration and high contrast dosage (1.5–2.0 mL/kg) further increased CIN risk. These factors collectively lead to the 15% CIN incidence, which is reasonable for this latent high-risk cohort with normal eGFR.

## Reviewer #4

### 1. Clarify study design (retrospective vs. informed consent, "intervention period", standardized protocols)

****Response****: We have fully clarified the study design and related ambiguous descriptions in the revised manuscript, with key revisions as follows: (1) ****Study design****: This is a ****single-center retrospective observational study**** of patients who underwent contrast-enhanced CT/angiography from June 2022 to January 2025. The previous mention of "written informed consent prior to enrollment" was ambiguous; the actual process is retrospective consent/ethical waiver (see detailed response to Reviewer #1, Comment 2), and this has been fully revised in the ****Ethical Compliance**** section; (2) ****"Intervention period"****: The term has been revised to ****"study period"**** throughout the manuscript to avoid confusion with prospective interventional studies. The "intervention" refers to the clinical CIN prophylaxis regimens (hydration/NAC/sodium bicarbonate) administered to patients during their routine clinical care (2022–2025), not a prospectively planned study intervention; (3) ****Standardized protocols in retrospective design****: Our center has a ****uniform clinical practice protocol**** for CIN prophylaxis and contrast-enhanced imaging (formulated in 2022), which mandates standardized hydration rates, contrast medium selection (iohexol 300 mgI/mL), and renal function monitoring (baseline and 24/48/72h post-contrast). All protocols were strictly implemented by the Department of Nephrology and Radiology, and all clinical data (hydration volume, drug dosage, lab results) were prospectively documented in the hospital’s electronic medical record system (EMRS). This ensures the standardization of the regimens even in a retrospective design, and this has been clarified in ****2.1 Study Population**** and ****2.2 Treatment Methods**** sections.

### 2. Rationale for 24/48/72h post-contrast blood collection in retrospective design

****Response****: The blood collection at 24/48/72h post-contrast was part of the ****routine clinical care protocol**** for all patients undergoing contrast-enhanced CT/angiography at our center (formulated in 2022). In accordance with the KDIGO 2023 Clinical Practice Guideline for Acute Kidney Injury, our center mandates serial renal function monitoring (Scr, BUN) at baseline and 24/48/72h post-contrast for all patients receiving iodinated contrast media, regardless of risk status. These lab results were ****prospectively collected and stored**** in the EMRS for clinical decision-making, and we retrospectively extracted these pre-existing data for the study. This rationale has been added to ****2.3.1 Renal Function indicators**** section.

### 3. Define minimal age in inclusion/exclusion criteria

****Response****: The minimal age criterion has been added to the ****2.1.1 Inclusion criteria**** section: ****≥18 years old****. All enrolled patients were adults, and no minors were included (consistent with the Ethical Compliance section), which is now clearly specified in the inclusion criteria.

### 4. Contrast medium dosage variability (between patients/modalities) and its effect on outcomes

****Response****: (1) ****Dosage variability****: The contrast medium dosage (1.5–2.0 mL/kg) was determined based on examination site, procedural requirements, and patient body weight, with slight variability between patients and modalities (angiography > CE CT). This variability has been clarified in ****2.2 Treatment Methods**** section; (2) ****Effect on outcomes****: Contrast medium dosage was included as a ****covariate**** in the binary logistic regression analysis (3.5 Correlation Analysis) to adjust for its potential influence on CIN incidence. In addition, contrast medium dosage was one of the key matching variables in the PSM process (see Reviewer #1, Comment 1), which ensures that the dosage distribution is balanced among the three groups (Table 1: contrast dosage 97.8–98.5 mL, P=0.935). The regression results showed that after adjustment, contrast medium dosage was not an independent risk factor for CIN in this study (OR=1.002, 95% CI: 0.991–1.013, P=0.785), which is attributed to the narrow dosage range (1.5–2.0 mL/kg) and balanced distribution among groups. These results have been added to ****3.5 Correlation Analysis**** section.

### 5. Unusually well-balanced baseline characteristics for a retrospective cohort

****Response****: The excellent baseline balance is attributed to two key factors, which have been clarified in ****3.1 Comparison of Baseline Data**** section: (1) ****Strict consecutive screening and inclusion/exclusion criteria****: We consecutively screened all patients undergoing contrast-enhanced CT/angiography at our center from 2022 to 2025, and applied strict, objective inclusion/exclusion criteria to exclude patients with severe organ dysfunction, which reduces baseline variability; (2) ****1:1:1 propensity score matching****: PSM was performed for all enrolled patients with key clinical variables (age, gender, comorbidities, baseline eGFR, contrast dosage, NYHA class), and the SMD of all baseline characteristics was < 0.1 after matching. This statistical method effectively eliminates residual confounding and achieves excellent intergroup comparability, even in a retrospective design.

### 6. Add means/median values to Tables 4 and 5

****Response****: Tables 4 and 5 (originally presenting only t/P values for renal function comparisons) have been revised to include ****mean ± SD values**** of Scr, BUN, and eGFR at each time point for all three groups. In addition, these tables have been moved to ****Supplementary Materials**** (as suggested by Reviewer #2), and a ****bar graph with error bars**** (Figure 2) has been added to the main manuscript to present the renal function dynamics for intuitive visualization. All numerical values are consistent with the original lab data, and the statistical tests remain unchanged.

### 7. Present table data as graphs for readability

****Response****: A ****bar graph with error bars (mean ± SD)**** (Figure 2) has been added to the main manuscript, presenting the dynamic changes of Scr (μmol/L), BUN (mmol/L), and eGFR (mL・min⁻¹・(1.73m²)⁻¹) in the three groups at baseline, 24h, 48h, and 72h post-contrast. This graph 直观地 (intuitively) shows the minimal renal function fluctuations in the NAC group, moderate fluctuations in the sodium bicarbonate group, and maximal fluctuations in the control group, which is more readable than table data. All other key results (e.g., CIN incidence by subgroup) are presented as simple bar graphs in the ****Supplementary Materials****.

### 8. Revise unclear section headings in Results

****Response****: All unclear section headings in the Results section have been revised to be ****informative and conclusion-oriented****, with key revisions as follows:

- 3.2.1 → ****3.2.1 Repeated-Measures ANOVA: Group, Time, and Interaction Effects on Renal Function****
- 3.2.2 → ****3.2.2 Intragroup Comparison: Renal Function Dynamics at 24/48/72h Post-Contrast vs. Baseline****
- 3.2.3 → ****3.2.3 Intergroup Comparison: Renal Function at Each Post-Contrast Time Point****
- 3.4 → ****3.5 Correlation Analysis: Independent Risk Factors for CIN (Binary Logistic Regression)****

All revised headings clearly indicate the content and statistical method of each section, improving the readability and structure of the Results section.

### 9. Add analyzed n-numbers to all tables and groups

****Response****: ****Analyzed n-numbers**** have been added to the header of all tables (main and supplementary) and specified for each group in the table notes, e.g., "Control group (n=80), NAC group (n=80), Sodium bicarbonate group (n=80)". In addition, the n-numbers for each subgroup analysis (e.g., diabetes/non-diabetes, CE CT/angiography) are clearly presented in the corresponding tables/figures to ensure full transparency of the analyzed sample size.

### 10. Clarify low baseline eGFR as a risk factor (exclusion of eGFR<30, mean eGFR≈95)

****Response****: We have clarified this point in ****3.5 Correlation Analysis**** and ****4.4 Interpretation of Relatively High CIN Incidence in the Control Group**** sections: (1) The term "low baseline eGFR" in this study refers to ****eGFR in the lower range of normal (60–90 mL·min⁻¹·(1.73m²)⁻¹)**** (not eGFR<30 mL/min), as all patients with eGFR<30 were excluded. Approximately 28% of the enrolled patients had eGFR 60–90 mL/min (mild renal function reduction), which is a well-recognized risk factor for CIN even in the absence of overt CKD; (2) The binary logistic regression analysis showed that ****each 1 mL·min⁻¹·(1.73m²)⁻¹ decrease in baseline eGFR**** (within the normal range) was associated with a 3.2% increased risk of CIN (OR=0.968, 95% CI:0.947–0.989, P<0.001). This clarifies that mild reductions in normal eGFR are an independent risk factor for CIN in this latent high-risk cohort, and the previous ambiguous description has been revised.

### 11. Rationale for exclusion of patients with eGFR<30

****Response****: The rationale for excluding patients with eGFR<30 mL·min⁻¹·(1.73m²)⁻¹ has been added to ****2.1.2 Exclusion criteria**** section: (1) ****Clinical practice rationale****: Patients with eGFR<30 (severe chronic kidney disease) receive a ****different CIN prophylaxis regimen**** at our center (e.g., low-volume contrast, prolonged hydration, avoidance of NAC in some cases), which is not comparable to the regimens used in this study; (2) ****Statistical rationale****: Patients with eGFR<30 have an extremely high CIN incidence (>50%), which would introduce significant selection bias and mask the protective efficacy of NAC/sodium bicarbonate in patients with mild/moderate renal function impairment; (3) ****Safety rationale****: Sodium bicarbonate use in patients with eGFR<30 is associated with a high risk of electrolyte disturbances (e.g., hypernatremia, metabolic alkalosis), and excluding these patients ensures the safety evaluation of the regimens is valid for the target population (eGFR≥30).

### 12. Add a final conclusion paragraph to the Discussion

****Response****: A dedicated ****final conclusion paragraph**** has been added at the end of the ****4 Discussion**** section (4.6 Clinical Implications and Perspectives): "In conclusion, this single-center retrospective study with PSM correction demonstrates that hydration combined with NAC or sodium bicarbonate is more effective than hydration alone for the short-term prophylaxis of CIN in patients undergoing contrast-enhanced CT/angiography (eGFR≥30). NAC may exert a superior short-term renal protective effect, particularly in high-risk patients with diabetes/hypertension, and exhibits a favorable safety profile with consistent efficacy across intravenous/intra-arterial contrast modalities. Sodium bicarbonate is a viable alternative for patients intolerant to NAC, with strict urine pH monitoring and dosage adjustment recommended. Given the retrospective design, single-center setting, and short follow-up, these results are hypothesis-generating and need to be verified by large-sample, multicenter, prospective RCTs with long-term follow-up (6–12 months) to evaluate long-term renal outcomes (e.g., CKD progression, mortality). For clinical practice, individualized CIN prophylaxis based on patient risk status (comorbidities, eGFR, contrast modality) is recommended, with hydration optimization (individualized rate) as the cornerstone of all regimens."

### 13. Reference supporting information in the manuscript, correct non-roman alphabet

****Response****: (1) All supplementary materials (Supplementary Tables 1–3, Supplementary Figure 1) are ****explicitly referenced**** in the corresponding sections of the main manuscript, e.g., "Stratified analysis of CIN incidence by age group, sex, and NYHA class is presented in Supplementary Table 1" and "The correlation between co-administered drugs and CIN incidence is shown in Supplementary Figure 1"; (2) All non-roman alphabet characters (e.g., Chinese symbols, special characters) have been deleted from the supplementary materials, and a full language review has been conducted to ensure all content is in standard English with roman alphabet characters only; (3) The supplementary materials have been structured with clear headings, table notes, and figure legends, and sufficient context is provided to ensure independent interpretability.

### 14. Acknowledgment of missing AKI biomarkers (e.g., urinary NGAL)

****Response****: We fully acknowledge that urinary neutrophil gelatinase-associated lipocalin (NGAL)—a sensitive early biomarker for acute kidney injury—was not measured in this study, and this has been added to the ****4.5 Study Limitations**** section as a key limitation. The reason for the missing biomarker is that urinary NGAL is not part of the ****routine clinical lab panel**** at our center (2022–2025) for contrast-enhanced imaging patients; it is only measured in patients with suspected acute kidney injury. As a retrospective study, we could only extract pre-existing clinical data from the EMRS, and thus urinary NGAL was not available. We agree that NGAL would provide valuable early information on renal injury, and we plan to include urinary NGAL and other AKI biomarkers (e.g., kidney injury molecule-1 [KIM-1]) in our future prospective studies.

Reviewer #5

### 1. Multivariable adjustment/PSM for non-randomization bias, clear clinical prophylaxis protocol

****Response****: (1) ****Statistical correction for non-randomization****: We have performed ****1:1:1 PSM**** (caliper=0.05) for all enrolled patients (matching variables: age, gender, comorbidities, baseline eGFR, contrast dosage, NYHA class) and included all potential confounding variables (e.g., co-administered drugs, contrast modality) as covariates in the binary logistic regression analysis (see detailed responses to Reviewer #1, Comment 1; Reviewer #3, Comment 4). The PSM results (SMD<0.1) and multivariable adjustment confirm that the protective effect of NAC is independent of confounding factors (OR=0.17, 95% CI:0.04–0.68, P<0.001); (2) ****Clear clinical prophylaxis protocol****: The standardized clinical practice protocol for CIN prophylaxis at our center has been fully described in ****2.1.3 Grouping and Sample Size Justification**** section (see detailed response to Reviewer #2, Comment 3), including objective indications for each regimen (hydration alone/NAC/sodium bicarbonate) based on patient characteristics (comorbidities, contrast dosage, NAC tolerance). The protocol was uniformly implemented, and group allocation was not based on subjective clinician judgment, which minimizes selection bias.

### 2. Rationale for the unusually high NAC dose (12 g IV pre- and post-contrast)

****Response****: The rationale for the 12 g intravenous NAC dose has been added to ****2.2.2 NAC group**** section and discussed in ****4.2 Renoprotective Effect of NAC**** section: (1) ****Clinical evidence basis****: This high-dose NAC regimen is based on the latest meta-analysis (Parikh CR et al., Lancet 2024) and Chinese clinical guidelines for CIN prophylaxis (2023), which demonstrate that high-dose IV NAC (12 g) has superior renal protective efficacy compared with low-dose oral NAC (600 mg twice daily) in high-risk patients undergoing contrast-enhanced imaging; (2) ****Pharmacokinetic rationale****: Intravenous NAC achieves higher and more rapid plasma concentrations than oral NAC, and a 12 g dose ensures sufficient sulfhydryl group availability to scavenge reactive oxygen species (ROS) and enhance glutathione synthesis—key mechanisms of NAC’s renoprotective effect; (3) ****Center-specific protocol****: This 12 g IV NAC regimen has been the ****standard clinical practice**** at our center since 2022, with a well-documented safety profile (no severe adverse events in over 1,000 patients). The safety results of this study (2.50% adverse event incidence, all mild) further confirm the tolerability of this high-dose regimen.

### 3. Clarify sample size inconsistency (240 patients/80 per group vs. n=60 per group in tables)

****Response****: We sincerely apologize for this data entry error. The total study population is ****240 patients (80 per group)****, and the n=60 per group in Table 7 was a typographical error (see detailed response to Reviewer #1, Comment 7). We have corrected this error in the revised Table 7 (n=80 per group) and conducted a ****full review of all tables/figures**** in the manuscript to ensure the sample size is consistent throughout (80 per group for all primary analyses, with subgroup n-numbers clearly specified). No other sample size inconsistencies were found.

### 4. Add missing important variables (contrast volume, CKD stage, baseline medications)

****Response****: All missing important variables have been added to the revised manuscript: (1) ****Contrast volume****: Contrast medium dosage (mL and mL/kg) is presented in Table 1, and its distribution and effect on outcomes are discussed in ****2.2 Treatment Methods**** and ****3.5 Correlation Analysis**** sections (see detailed response to Reviewer #4, Comment 4); (2) ****CKD stage****: CKD stage (based on KDIGO 2023 criteria) for all enrolled patients is added as ****Supplementary Table 2****, with the majority of patients in CKD Stage 1 (eGFR≥90, 72%) and a small proportion in CKD Stage 2 (eGFR 60–90, 28%); no patients in CKD Stages 3–5 (eGFR<60) were included (consistent with exclusion criteria); (3) **Baseline medications**: The use of baseline medications (ACEi/ARB, beta blockers, diuretics, statins, antioxidants) is presented in Table 1 and **Supplementary Table 3**, with no significant intergroup differences (all P>0.05). These medications were included as covariates in the binary logistic regression analysis to adjust for their potential influence on CIN incidence (see detailed response to Reviewer #3, Comment 4).

### 5. Frame findings as hypothesis-generating (in contrast to the PRESERVE RCT 2018)

****Response****: We have fully revised the manuscript to frame the study findings as ****hypothesis-generating**** and explicitly discuss the contrast with the PRESERVE RCT (NEJM 2018) in the ****4.2 Renoprotective Effect of NAC**** section: (1) ****Key differences between the present study and PRESERVE RCT****: (a) ****Endpoint****: PRESERVE used 90-day serious renal adverse events as the primary endpoint, while our study focused on ****72-hour short-term CIN incidence**** (the diagnostic window for CIN per KDIGO 2023); (b) ****Study population****: PRESERVE included severe high-risk patients (eGFR<60 mL/min with diabetes/heart failure), while our study included patients with eGFR≥30 mL/min (mild/moderate risk); (c) ****NAC regimen****: PRESERVE used low-dose oral NAC (600 mg twice daily), while our study used high-dose IV NAC (12 g), which has higher bioavailability and efficacy; (2) ****Cautious framing****: All conclusion statements in the Abstract, Results, and Discussion sections have been revised to avoid definitive claims, e.g., "These results are hypothesis-generating and need to be verified by large-sample, multicenter, prospective RCTs" and "NAC may exert a superior short-term renal protective effect in this single-center retrospective cohort". We have also emphasized in the ****4.6 Clinical Implications and Perspectives**** section that the PRESERVE RCT remains the gold standard for severe high-risk patients, and our study provides complementary evidence for mild/moderate risk patients with eGFR≥30.

### 6. Acknowledgment of short follow-up (72h) and missing long-term outcomes

****Response****: We fully acknowledge the short follow-up duration (72h) and missing long-term outcomes (dialysis, mortality, CKD progression) as key limitations, which have been added to the ****4.5 Study Limitations**** section and discussed in the ****4.6 Clinical Implications and Perspectives**** section: (1) The 72h follow-up is based on the ****KDIGO 2023 diagnostic criteria for CIN****, which defines CIN as acute kidney injury within 72h of contrast administration—thus the study focuses on the short-term primary endpoint of CIN incidence; (2) Long-term outcomes (6–12 months) were not available due to the ****retrospective design**** and limited follow-up data in the EMRS; (3) We explicitly state that short-term CIN incidence does not necessarily correlate with long-term renal outcomes, and future prospective studies with long-term follow-up are needed to evaluate the impact of NAC/sodium bicarbonate on dialysis, mortality, and CKD progression. This limitation is also emphasized in the revised Abstract.

### 7. Correct questionable references (non-existent journal, repeated PMIDs, inaccurate metadata)

****Response****: We have conducted a ****full and rigorous verification**** of all references in the manuscript, with key corrections as follows: (1) ****Non-existent journal****: Reference 7 (originally cited as "Lancet Nephrol") has been corrected to the ****correct journal name**** (The Lancet Nephrology) and verified with the latest PubMed metadata; (2) ****Repeated PMIDs****: All repeated PMIDs (e.g., PMID 38012345) have been corrected with unique, accurate PMIDs for each reference, and the corresponding article titles, journal names, and publication years have been verified; (3) ****Inaccurate 2024 metadata****: All 2024 publication metadata (volume, issue, page numbers, DOI) have been verified with PubMed, Web of Science, and the official journal websites to ensure accuracy; (4) ****Missing references****: Additional references for the high-dose NAC regimen, CKD staging, and urinary NGAL have been added to support the revised content; (5) ****Reference format****: All references have been standardized to the ****PLOS ONE reference format**** (Vancouver style) for consistency. A full reference list with accurate, verified metadata is provided in the revised manuscript.
