# Supplementary material for: Sodium Bicarbonate versus N-Acetylcysteine plus hydration versus hydration alone for preventing contrast-associated acute kidney injury: A single-center retrospective analysis with propensity score matching
Source: PLoS One. 2026 Jul 9;21(7):e0353461. doi: 10.1371/journal.pone.0353461 (PMC13349148; doi:10.1371/journal.pone.0353461)
Supplement: Attachment — Submitted filename: Response_to_Reviewers_auresp_2.docx [file pone.0353461.s004.docx]

**Response to Editorial Instructions**

**Manuscript ID:** PONE-D-26-01652R1

**Title:** Sodium Bicarbonate versus N-Acetylcysteine plus Hydration vs Hydration Alone for Preventing Contrast-Associated Acute Kidney Injury: A Single-Center Retrospective Analysis with Propensity Score Matching

Dear Dr. Li Yang and the esteemed reviewers,

Thank you very much for your careful and constructive comments on our manuscript. We have carefully addressed all the points raised and revised the manuscript accordingly. All changes are highlighted using track changes in the "Revised Manuscript with Track Changes" file, and a clean version is provided as "Manuscript". Below is our point-by-point response to each comment.

We believe the revised manuscript has been significantly improved and now fully meets the publication criteria of PLOS ONE. We look forward to your favorable consideration.

Sincerely, Dr. Ying-Huan Ma and co-authors Corresponding Author Department of Nephrology, The Air Force Hospital of Northern Theater PLA Email: [952006555@qq.com](mailto:952006555@qq.com)

# Response to Reviewers

# **Response to Reviewer #1**

## **Comment:** All comments have been addressed.

## **Response:** We sincerely appreciate your recognition and support of our work. We have carefully reviewed the entire manuscript again to ensure no typos or formatting errors remain, and all previous revisions are retained in the current version.

## **Response to Reviewer #2**

**Comment 1:** Please add to section "2.6 Sample Size Calculation" the comments placed in Point-by-Point Responses to Reviewers’ Comments, specifically the power size recalculation.

**Response:** Thank you for this important suggestion. We have added detailed post-hoc power analysis results to Section 2.6 Sample Size Calculation, including the statistical power for the primary outcome and subgroup analyses, as well as the rationale for not including an attrition rate. The revised section is provided below (see Manuscript, Section 2.6, Lines 265–289).

**Comment 2:** Add footnotes to Table 2.

**Response:** We have added comprehensive footnotes to Table 2, explaining the Greenhouse-Geisser correction, the definition of partial η² (effect size), and the interpretation of effect size magnitudes. The revised Table 2 is included in the manuscript (see Manuscript, Table 2, Lines 484–488).

**Comment 3:** Figure 2 and Supplementary information is mentioned in Responses to Reviewers but not localized in the current version.

**Response:** We apologize for this oversight. We have:Added Figure 2 (Dynamic changes in serum creatinine and eGFR over 72 hours in the three groups) to the Results section, with corresponding citations in the text (see Manuscript, Section 3.2.2, Lines489–509).

## **Response to Reviewer #3**

**Comment:** Solutions for removing of confounding factors have not been addressed clearly, especially about selection biases and other confounders.

**Response:** Thank you for this critical comment. We have comprehensively revised the manuscript to clarify our strategies for controlling confounding factors and selection bias, with revisions in three key sections:

**1.Section 2.4 Group Allocation (Lines 228–244):** Added detailed explanation of how our institutional protocol-based grouping minimized physician-related selection bias, and why we included all core baseline risk factors in the PSM model to address residual confounding from risk stratification.

**2.Section 2.5 Propensity Score Matching (Lines 245–264):** Expanded the description of PSM methodology, including the rationale for choosing 1:1:1 nearest-neighbor matching with a 0.05 caliper, the use of standardized mean difference (SMD) for balance assessment, and verification of the common support domain.

**3.Section 4.6 Study Limitations (Lines 709–749):** Explicitly acknowledged the potential for residual confounding from unmeasured variables (e.g., baseline volume status, contrast injection rate) and explained how our rigorous study design and PSM adjustment minimized this risk.

All revisions are highlighted in the track changes version, and we believe they fully address your concerns about confounding and selection bias.
