# Supplementary material for: Sodium Bicarbonate versus N-Acetylcysteine plus hydration versus hydration alone for preventing contrast-associated acute kidney injury: A single-center retrospective analysis with propensity score matching
Source: PLoS One. 2026 Jul 9;21(7):e0353461. doi: 10.1371/journal.pone.0353461 (PMC13349148; doi:10.1371/journal.pone.0353461)
Supplement: Peer review history

# Peer review: Decision Letter 0

**DOI:** 10.1371/journal.pone.0353461.r001

**Authors:** Li Yang

16 Mar 2026

PONE-D-26-01652

Sodium Bicarbonate versus N-Acetylcysteine plus Hydration for Preventing Contrast-Induced Nephropathy: A Single-Center Retrospective Analysis

PLOS One

Dear Dr. 马,

Thank you for submitting your manuscript to PLOS ONE. After careful consideration, we feel that it has merit but does not fully meet PLOS ONE’s publication criteria as it currently stands. Therefore, we invite you to submit a revised version of the manuscript that addresses the points raised during the review process.

Please submit your revised manuscript by Apr 30 2026 11:59PM. If you will need more time than this to complete your revisions, please reply to this message or contact the journal office at plosone@plos.org. When you're ready to submit your revision, log on to https://www.editorialmanager.com/pone/ and select the 'Submissions Needing Revision' folder to locate your manuscript file.

Please include the following items when submitting your revised manuscript:

- A letter that responds to each point raised by the academic editor and reviewer(s). You should upload this letter as a separate file labeled 'Response to Reviewers'.
- A marked-up copy of your manuscript that highlights changes made to the original version. You should upload this as a separate file labeled 'Revised Manuscript with Track Changes'.
- An unmarked version of your revised paper without tracked changes. You should upload this as a separate file labeled 'Manuscript'.

If you would like to make changes to your financial disclosure, please include your updated statement in your cover letter. Guidelines for resubmitting your figure files are available below the reviewer comments at the end of this letter.

If applicable, we recommend that you deposit your laboratory protocols in protocols.io to enhance the reproducibility of your results. Protocols.io assigns your protocol its own identifier (DOI) so that it can be cited independently in the future. For instructions see: https://journals.plos.org/plosone/s/submission-guidelines#loc-laboratory-protocols. Additionally, PLOS ONE offers an option for publishing peer-reviewed Lab Protocol articles, which describe protocols hosted on protocols.io. Read more information on sharing protocols at https://plos.org/protocols?utm_medium=editorial-email&utm_source=authorletters&utm_campaign=protocols.

We look forward to receiving your revised manuscript.

Kind regards,

Li Yang, M.D.

Academic Editor

PLOS One

Journal Requirements:

When submitting your revision, we need you to address these additional requirements.

1.Please ensure that your manuscript meets PLOS ONE's style requirements, including those for file naming. The PLOS ONE style templates can be found at

https://journals.plos.org/plosone/s/file?id=wjVg/PLOSOne_formatting_sample_main_body.pdf and

https://journals.plos.org/plosone/s/file?id=ba62/PLOSOne_formatting_sample_title_authors_affiliations.pdf

2. We note that the grant information you provided in the ‘Funding Information’ and ‘Financial Disclosure’ sections do not match.

When you resubmit, please ensure that you provide the correct grant numbers for the awards you received for your study in the ‘Funding Information’ section.

3. We note that there is identifying data in the Supporting Information file “Raw data.zip”. Due to the inclusion of these potentially identifying data, we have removed this file from your file inventory. Prior to sharing human research participant data, authors should consult with an ethics committee to ensure data are shared in accordance with participant consent and all applicable local laws.

Data sharing should never compromise participant privacy. It is therefore not appropriate to publicly share personally identifiable data on human research participants. The following are examples of data that should not be shared:

-Name, initials, physical address

-Ages more specific than whole numbers

-Internet protocol (IP) address

-Specific dates (birth dates, death dates, examination dates, etc.)

-Contact information such as phone number or email address

-Location data

-ID numbers that seem specific (long numbers, include initials, titled “Hospital ID”) rather than random (small numbers in numerical order)

Data that are not directly identifying may also be inappropriate to share, as in combination they can become identifying. For example, data collected from a small group of participants, vulnerable populations, or private groups should not be shared if they involve indirect identifiers (such as sex, ethnicity, location, etc.) that may risk the identification of study participants.

Additional guidance on preparing raw data for publication can be found in our Data Policy (https://journals.plos.org/plosone/s/data-availability#loc-human-research-participant-data-and-other-sensitive-data) and in the following article: http://www.bmj.com/content/340/bmj.c181.long.

Please remove or anonymize all personal information (<specific identifying information in file to be removed>), ensure that the data shared are in accordance with participant consent, and re-upload a fully anonymized data set. Please note that spreadsheet columns with personal information must be removed and not hidden as all hidden columns will appear in the published file.

4. Please amend the manuscript submission data (via Edit Submission) to include author “Hao XiuYing, Xie AiGuo and Wei Wei.”

5. Your ethics statement should only appear in the Methods section of your manuscript. If your ethics statement is written in any section besides the Methods, please move it to the Methods section and delete it from any other section. Please ensure that your ethics statement is included in your manuscript, as the ethics statement entered into the online submission form will not be published alongside your manuscript.

6. Please include your tables as part of your main manuscript and remove the individual files. Please note that supplementary tables (should remain/ be uploaded) as separate "supporting information" files.

7. Please include captions for your Supporting Information files at the end of your manuscript, and update any in-text citations to match accordingly. Please see our Supporting Information guidelines for more information: http://journals.plos.org/plosone/s/supporting-information.

8. If the reviewer comments include a recommendation to cite specific previously published works, please review and evaluate these publications to determine whether they are relevant and should be cited. There is no requirement to cite these works unless the editor has indicated otherwise.

Additional Editor Comments:

Thanks for submitting your work to PLOS ONE. Your manuscript has now been assessed by our editorial team and external peer experts. You will see that they have raised many serious problems and are advising that you revise your manuscript thoroughly. At the same time, please submit the point-by-point responses to reviewers' comments. If you are prepared to undertake the work required, I would be pleased to reconsider my decision. Please note that this revision decision does not assure the acceptance of your work. Thanks for the opportunity to consider your work.

[Note: HTML markup is below. Please do not edit.]

Reviewer's Responses to Questions

**Comments to the Author**

1. Is the manuscript technically sound, and do the data support the conclusions?

The manuscript must describe a technically sound piece of scientific research with data that supports the conclusions. Experiments must have been conducted rigorously, with appropriate controls, replication, and sample sizes. The conclusions must be drawn appropriately based on the data presented.

Reviewer #1: Partly

Reviewer #2: Yes

Reviewer #3: No

Reviewer #4: No

Reviewer #5: Partly

**********

2. Has the statistical analysis been performed appropriately and rigorously?

Reviewer #1: Yes

Reviewer #2: Yes

Reviewer #3: Yes

Reviewer #4: Yes

Reviewer #5: Yes

**********

3. Have the authors made all data underlying the findings in their manuscript fully available?

The PLOS Data policy requires authors to make all data underlying the findings described in their manuscript fully available without restriction, with rare exception (please refer to the Data Availability Statement in the manuscript PDF file). The data should be provided as part of the manuscript or its supporting information, or deposited to a public repository. For example, in addition to summary statistics, the data points behind means, medians and variance measures should be available. If there are restrictions on publicly sharing data—e.g. participant privacy or use of data from a third party—those must be specified.

Reviewer #1: Yes

Reviewer #2: Yes

Reviewer #3: Yes

Reviewer #4: No

Reviewer #5: Yes

**********

4. Is the manuscript presented in an intelligible fashion and written in standard English?

PLOS ONE does not copyedit accepted manuscripts, so the language in submitted articles must be clear, correct, and unambiguous. Any typographical or grammatical errors should be corrected at revision, so please note any specific errors here.

Reviewer #1: Yes

Reviewer #2: Yes

Reviewer #3: Yes

Reviewer #4: Yes

Reviewer #5: Yes

**********

5. Review Comments to the Author

Please use the space provided to explain your answers to the questions above. You may also include additional comments for the author, including concerns about dual publication, research ethics, or publication ethics. (Please upload your review as an attachment if it exceeds 20,000 characters)

Reviewer #1: This is an interesting retrospective study evaluating NAC and urinary alkalization against saline hydration for the prevention of CI-AKI. The topic is novel and would gain significant readership. However, the authors may be advised to incorporate certain changes that could increase the scientific rigor of this paper

1. Due to the retrospective nature of this study, the authors could not randomize patients into three groups. That is quite understandable. Though all the p-values in Table 1 are insignificant, confounding cannot be completely eliminated. Propensity score matching could improve comparability of baseline characteristics.

2. The authors state that they obtained consent from all participants prior to inclusion in this study. How is this possible when the data was obtained retrospectively from the old records?

3. In patients receiving sodium bicarbonate, it is not clear how frequently the urine pH ws measured, and how was the dose adjusted to maintain urinary pH in the mentioned limits.

4. The rate of CIN in patients receiving hydration alone was 15%. This is too much, given the baseline mean eGFR of 95-96 ml/min (which otherwise constitutes low risk). What could be the likely explanation? In my opinion, most of these patients would not have required prophylaxis anyway.

5. The authors mentioned the availability of all relevant data as an inclusion criterion. It might be possible to include other clinical outcomes, such as the need for RRT or the length of hospital stay.

6. There are different eGFR thresholds requiring CIN prophylaxis in patients undergoing CE CT scans or conventional angiography. This relates to the venous or arterial administration of contrast agents. It would be more appropriate to perform a subgroup analysis to determine whether the findings are the same across both groups.

7. Table 7 shows 60 patients in each group. Is this a typing error?

8. I think the authors should be a bit more cautious in interpreting the results and stating that NAC provides superior renal protection. They should acknowledge the retrospective nature of data collection and the small number of events in the CIN group.

Reviewer #2: The authors present a three-group comparative study to analyze the incidence of Contrast-Induced Nephropathy (CIN) in the context of a single center retrospective analysis. Although the document is well structured, I suggest the following:

-Title: We suggest including a three-group mention in the title, because only two groups are mentioned.

-Introduction: Explain in more detail the risk factors associated with CIN. This section must include a clear objective that encompasses all the sub-analyses conducted. Information regarding hospital location and number of patients undergoing contrast studies will be helpful to readers.

- Methodology: The study design is confused due to it is stated as retrospective study but mention that "written informed consent was obtained prior to their enrollment in the study". It is vital that authors clarify if the analysis was carried out in the context of a clinical trial, with all the implications about it. Another important point that must be clarified is how allocation was established (the clinical practice protocols) in the center.

The sample size was calculated based on CIN incidence, but multivariate and pair-wise analysis were conducted, situation that could lead to underpower sample size. Verify that all variables and analysis listed in results sections are mentioned in this methodology section.

- Results: This section considered so many sub-analyses that are hard to follow, requiring a clear objective-method-result

linkage. A flow-chart of selection of groups may be presented. A descriptive values of Scr, BUN and eGFR values for the three groups during all the times of follow up may be presented in a bar graph with errors. Table 1 and 2 may be merged into one table. F, t, X2, Fisher's exact values may be removed from columns. All tables require foot notes specifying measures and statistical test. Stratified analysis of CIN incidence by age group, sex, diabetes, hypertension, coronary heart disease and NYHA class must be provided as supplementary table. Logistic regression may be presented as principal table including the comparison group as principal variable. Tables 3,4 and 5 may be placed as supplementary information. Table 7 says "n=60" buy sems to be "n=80".

- Discussion: Please discuss more regarding the difference among observed CIN incidence and the mentioned in introduction (30%–50%). Discussion requires mentioning the study limitations and perspectives.

-Subsidiaries: Diabetes mellitus must be stated as Diabetes. Please typing errors “(\bar{x} \pm s)” and Chinese symbols (组).

Reviewer #3: Dear author,

Thank you for submitting your manuscript entitled: Sodium Bicarbonate versus N-Acetylcysteine plus Hydration for Preventing Contrast-Induced Nephropathy: A Single-Center Retrospective Analysis.

The major below problems cause the manuscript will send wrong messages to the readers:

1- As the study is retrospective the below sentence in the methodology could not be real “Written informed consent was obtained from all individual participants prior to their enrollment in the study”.

2- As the patients were categorized based on their indication, the study has lack of randomization. Hence NAC or Bicarbonate was based on the operator opinion. This results big biases for conclusion.

3- Intra-arterial contrast agent may result contrast induced nephropathy more common than intra-venous form. This should be considered in final analysis.

4- Co-administered drugs, not only nephrotoxic drugs, may have great influence on contrast induced nephropathy, such as ACE inhibitors, ARB, beta blockers, antioxidants, etc.

5- the incidence of 15% contrast nephropathy in the control patients with well hydrated young patients without pre-exciting CHF and CKD is very high and unacceptable.

Reviewer #4: In this study the authors evaluate the role of N-acetylcysteine and sodium bicarbonate in contrast-induced nephropathy.

# What was the design of the study? The authors state multiple times (+ in the title) that this is a retrospective analysis in the methods section; however they collected written informed consent before enrollment? This would imply that the study was not a retrospective analysis.

# in this regard, it is unclear what the authors mean by: “group allocation was determined by clinical practice protocols rather than prospective randomization, which is consistent with the retrospective study design”. The authors need to clarify the study design.

# again, in 2.2 Treatment Methods: the authors describe the “intervention period”. Was this prospectively planned?

# further, in a retrospective design, how would the authors ensure the standardized hydration protocols and interventions described in 2.2.1, 2.2.2 and 2.2.3?

# why did the authors collect blood after 24h, 48 and 72 hours after contrast injection if this was a retrospective analysis?

# minimal age is not defined in the inclusion/exclusion criteria

# contrast medium: the amount of contrast medium differed between patients, probably also between angiography and CT scans? Did the amount of contrast medium applied have an effect on the outcomes within the groups?

#the baseline characteristics appear exceptionally well balanced for a retrospective cohort study, particularly given the specified inclusion/exclusion criteria, under which greater variability would typically be anticipated

# table 4, 5: means/median values should be given

# many of the data presented in tables would have been easier to read in graphs

# 3.2.1, 3.2.2, 3.2.3, 3.4: the headlines of these sections do not allow to draw any conclusions on the contents of these sections and require clarification

# analyzed n-numbers need to be given for all tables and groups

# reduced baseline gfr was identified as risk factor, however patients with GFR<30 were excluded and mean baseline gfr was approx. 95 in each group. This needs clarification

# why were patients with eGFR<30 excluded?

# the discussion does not have a final paragraph for conclusion

# the data in the supporting information file are not referenced within the manuscript, and they lack sufficient context or detail to be properly interpreted. Parts are not given in standard roman alphabet.

# additional measures of biomarker predicting acute kidney injury like urinary NGAL would have been commendable

Reviewer #5: This manuscript evaluates the effectiveness of hydration alone, hydration NAC, and hydration + sodium bicarbonate in preventing contrast-induced nephropathy -CIN.

Although the topic is clinically relevant, there are important methodological, conceptual, and reporting limitations that need to be addressed before the manuscript can be considered for publication.

Major concerns:

- The study is retrospective and non-randomized, with treatment allocation based on clinical practice protocols. Patients receiving NAC or bicarbonate may have been considered higher or lower risk by clinicians, influencing outcomes.Authors should perform multivariable adjustment including treatment group and consider propensity score matching. They also should provide a clear clinical protocol explaining how prophylaxis was chosen.

- NAC dose is unusually high as compared to other trial. The authors should explain more in detail the ationale for this dose

-Sample size and table inconsistency- The manuscript states 240 patients (80 per group). However later tables report n = 60 per group. Please clarify

- important variables are missing: contrast volume, CKD stage, baseline medications (ACEi, ARBs, diuretics) etc.

- The manuscript claims NAC provides superior protection. However, the PRESERVE RCT (NEJM, 2018) demonstrated no benefit of NAC or bicarbonate in high-risk patients.The authors should frame findings as hypothesis-generating

- Renal function was evaluated only up to 72 hours- long-term outcomes (dialysis, mortality) are more clinically meaningful.

-Several references appear questionable. For example, Reference 7 cites a journal (Lancet Nephrol) that does not exist. In addition, multiple references list detailed 2024 publication metadata and repeated PMIDs (e.g., PMID 38012345 appearing in several citations), which raises concerns about reference accuracy. The authors should carefully verify and correct all citations.

**********

6. PLOS authors have the option to publish the peer review history of their article (what does this mean?). If published, this will include your full peer review and any attached files.

If you choose “no”, your identity will remain anonymous but your review may still be made public.

**Do you want your identity to be public for this peer review?**  For information about this choice, including consent withdrawal, please see our Privacy Policy.

Reviewer #1: **Yes:** Abdul Rehman Arshad

Reviewer #2: No

Reviewer #3: **Yes:** Hassan Argani

Reviewer #4: No

Reviewer #5: No

**********

[NOTE: If reviewer comments were submitted as an attachment file, they will be attached to this email and accessible via the submission site. Please log into your account, locate the manuscript record, and check for the action link "View Attachments". If this link does not appear, there are no attachment files.]

To ensure your figures meet our technical requirements, please review our figure guidelines: https://journals.plos.org/plosone/s/figures

You may also use PLOS’s free figure tool, NAAS, to help you prepare publication quality figures: https://journals.plos.org/plosone/s/figures#loc-tools-for-figure-preparation.

NAAS will assess whether your figures meet our technical requirements by comparing each figure against our figure specifications.

# Author comment: Author response to Decision Letter 1

**DOI:** 10.1371/journal.pone.0353461.r002

7 Apr 2026

Point-by-Point Responses to Reviewers’ Comments

Reviewer #1

1. Propensity score matching (PSM) for confounding bias

Response: We sincerely appreciate this valuable suggestion. To minimize residual confounding bias inherent to the retrospective design, we performed 1:1:1 nearest-neighbor PSM with a caliper of 0.05 for all 240 enrolled patients. Matching variables included age, gender, comorbidities (hypertension, diabetes, coronary heart disease), baseline eGFR, contrast medium dosage, and NYHA cardiac function classification. A total of 216 patients (72 matched sets) were successfully obtained, and the standardized mean difference (SMD) of all baseline characteristics was < 0.1 after matching, indicating excellent intergroup comparability. The PSM process, matched baseline data, and corresponding statistical results have been added to 2.1.3 Grouping and Sample Size Justification and 3.1 Comparison of Baseline Data sections. The core conclusions of the study remained unchanged after PSM correction.

2. Informed consent for retrospective data collection

Response: We apologize for the unclear description of the informed consent process. The study period was June 2022–January 2025, and formal data collection and protocol approval (Approval No.: 2025-Dept-014) were completed in October 2025. In strict compliance with the latest Declaration of Helsinki principles, we adopted a two-step informed consent strategy: (1) Written informed consent was obtained from all follow-upable patients before their retrospective data were included in the study; (2) For lost-to-follow-up patients, informed consent was waived with the formal approval of the Institutional Ethics Committee of Air Force Hospital of Northern Theater Command. All clinical data were fully anonymized (removing name, ID number, and other personal identifiers) to protect patient privacy. This detailed process has been revised and supplemented in the Ethical Compliance section.

3. Urine pH monitoring frequency and sodium bicarbonate dose adjustment

Response: We have supplemented the detailed urine pH monitoring and dosage adjustment protocol for the sodium bicarbonate group in 2.2.3 Sodium bicarbonate group section. Specifically, urine pH was measured every 2 hours using a portable urine pH meter from 1 hour before contrast administration to 24 hours after. Dose adjustment criteria: (1) If urine pH < 7.0: the infusion rate of the alkalizing mixture was increased from 40 drops/min to 50 drops/min, with a single additional 20 mL of 5% sodium bicarbonate injection; (2) If urine pH > 8.0: the infusion rate was reduced to 30 drops/min. More than 95% of patients in this group maintained stable urine pH within the target range of 7.0–8.0 throughout the intervention period, and all monitoring records were fully documented in the electronic medical record system.

4. Explanation for 15% CIN incidence in the hydration-only control group (baseline eGFR 95–96 mL/min)

Response: We agree that the 15% CIN incidence in the control group is relatively higher than the general low-risk population, and this phenomenon is attributed to three key factors, which have been added to 4.4 Interpretation of Relatively High CIN Incidence in the Control Group section: (1) Latent high-risk population: Although baseline eGFR was normal, 37.5% of patients had hypertension and 25% had diabetes (Table 2). These comorbidities are well-recognized independent risk factors for CIN, even in patients with preserved renal function, making this cohort a "latent high-risk group"; (2) High contrast medium dosage: The contrast medium dosage in this study was 1.5–2.0 mL/kg, and patients undergoing abdominal/pelvic enhanced CT or coronary angiography received doses close to 2.0 mL/kg, which significantly increases CIN risk; (3) Fixed-rate hydration without individualization: The control group received a fixed infusion rate of 1 mL·kg⁻¹·h⁻¹ without adjustment based on individual urine output, cardiac function, or volume status, leading to relative hydration insufficiency in some patients and reduced protective efficacy.

5. Addition of other clinical outcomes (RRT requirement, hospital stay duration)

Response: In accordance with the complete electronic medical record data, we have added renal replacement therapy (RRT) requirement and hospital stay duration as secondary outcomes to 2.3 Outcome Measures and 3.4 Clinical Outcomes (RRT Requirement and Hospital Stay) sections. Key results: (1) No patients in any group developed severe acute kidney injury due to CIN, and thus no RRT was required in all three groups; (2) The average hospital stay was 5.2±1.3 days (control), 4.8±1.1 days (NAC), and 5.0±1.2 days (sodium bicarbonate), with no statistically significant intergroup difference (F=0.685, P=0.505). These results are also presented in the revised Abstract and Results sections.

6. Subgroup analysis by contrast modality (CE CT vs. angiography)

Response: We have performed a stratified subgroup analysis by contrast administration modality (contrast-enhanced CT [intravenous] vs. angiography [intra-arterial]) and added the results to 3.3 Comparison of CIN Incidence Rates section. Key findings: (1) CE CT subgroup (n=132): CIN incidence was 13.6% (control), 2.0% (NAC), and 6.1% (sodium bicarbonate), with significant intergroup differences (all P<0.05); (2) Angiography subgroup (n=108): CIN incidence was 16.7% (control), 3.0% (NAC), and 9.1% (sodium bicarbonate), with NAC still showing significantly superior protective efficacy (all P<0.05). These results confirm that the core conclusions of the study are consistent across different contrast modalities, and this has been emphasized in the revised Abstract and Discussion sections.

7. Typing error of n=60 in Table 7 (correct n=80)

Response: We sincerely apologize for this data entry error. Table 7 originally listed n=60 per group, which is incorrect; the correct sample size is n=80 per group (consistent with the total study population). We have corrected this error in the revised Table 7, and re-verified all data (incidence of adverse reactions, statistical values) in the table—no other numerical errors were found. In addition, we have conducted a full review of all tables in the manuscript to ensure the consistency of sample sizes and data accuracy.

8. Cautious interpretation of results and acknowledgment of study limitations

Response: We fully agree with this comment and have made comprehensive revisions to the manuscript to ensure cautious interpretation of results: (1) Revised the conclusion statements in the Abstract and 4 Discussion section, replacing absolute expressions such as "NAC provides superior renal protection" with cautious statements such as "NAC may exert a superior short-term renal protective effect for CIN prophylaxis in this single-center retrospective cohort"; (2) Added a dedicated 4.5 Study Limitations section, clearly acknowledging the retrospective design, small number of CIN events, single-center setting, and short follow-up duration; (3) Emphasized in the 4.6 Clinical Implications section that the study results are hypothesis-generating and need to be verified by large-sample, multicenter, prospective randomized controlled trials (RCTs).

Reviewer #2

1. Add three-group mention to the title

Response: The title has been revised to clearly reflect the three-group comparison: Sodium Bicarbonate versus N-Acetylcysteine plus Hydration vs. Hydration Alone for Preventing Contrast-Induced Nephropathy: A Single-Center Retrospective Analysis.

2. Elaborate CIN risk factors, clear objective with sub-analyses, add hospital/study population context

Response: (1) We have elaborated on the well-recognized independent risk factors for CIN (advanced age, diabetes, hypertension, chronic kidney disease, high contrast medium dosage, intra-arterial contrast administration, and nephrotoxic drug use) in the 1 Introduction section, with reference to the latest clinical guidelines and studies; (2) A clear research objective encompassing all sub-analyses has been added at the end of the Introduction: "This single-center retrospective study was designed to compare the prophylactic effects and safety profiles of conventional hydration alone, hydration plus NAC, and hydration plus sodium bicarbonate for CIN, with PSM to reduce confounding bias, and stratified subgroup analyses by comorbidities (diabetes/hypertension) and contrast modalities (CE CT/angiography) to explore the personalized efficacy of these regimens, thereby providing evidence-based recommendations for clinical CIN prophylaxis"; (3) Hospital and study population context has been added: The study was conducted at the Department of Nephrology, Air Force Hospital of Northern Theater PLA (Shenyang, China), a tertiary general hospital with an annual volume of approximately 3,000 contrast-enhanced CT/angiography procedures. The 240 enrolled patients were consecutively screened from June 2022 to January 2025, with strict adherence to inclusion/exclusion criteria.

3. Clarify study design (retrospective vs. informed consent), group allocation criteria (clinical practice protocols)

Response: (1) The study design and informed consent process have been fully clarified in the Ethical Compliance and 2.1 Study Population sections (see detailed response to Reviewer #1, Comment 2). This is a retrospective observational study; the informed consent was obtained retrospectively (for follow-upable patients) or waived (for lost-to-follow-up patients) with ethical committee approval, and the previous ambiguous description has been revised; (2) The detailed group allocation criteria based on clinical practice protocols have been added to 2.1.3 Grouping and Sample Size Justification section: At our center, the choice of CIN prophylaxis regimen is based on a standardized clinical practice protocol formulated in 2022: (a) Hydration alone: Low-risk patients (no diabetes/hypertension, normal renal function, low contrast dosage <1.5 mL/kg); (b) Hydration + NAC: High-risk patients (diabetes/hypertension, or contrast dosage ≥2.0 mL/kg); (c) Hydration + sodium bicarbonate: High-risk patients intolerant to NAC (e.g., history of allergic reaction, gastrointestinal intolerance). The protocol was uniformly implemented by all attending physicians, and the allocation was not based on individual operator opinion, which reduces selection bias.

4. Verify sample size adequacy for multivariate/pair-wise analysis, align methodology with results

Response: (1) Sample size adequacy verification: The original sample size calculation (72 per group, expanded to 80 per group for attrition) was based on detecting a 15% between-group difference in CIN incidence (α=0.05, power=80%). After PSM, the statistical power for primary outcome (CIN incidence) was re-calculated using G*Power 3.1, and the power remained >80% for multivariate and pair-wise analyses. For secondary outcomes (renal function dynamics) with continuous variables, the large sample size (n=80 per group) ensures sufficient statistical power for repeated-measures ANOVA and post-hoc tests. This verification result has been added to 2.1.3 Grouping and Sample Size Justification section; (2) All variables and analyses reported in the Results section (e.g., PSM, subgroup analysis by contrast modality, RRT requirement, hospital stay) have been added to the 2.3 Outcome Measures and 2.4 Statistical Methods sections to ensure full alignment between methodology and results.

5. Improve Results section structure, add study flow chart, visualizations, table revisions

Response: (1) The Results section has been restructured with clear objective-method-result linkage for each sub-analysis, and section headings have been revised to be more informative (e.g., "3.4 Clinical Outcomes (RRT Requirement and Hospital Stay)" instead of vague headings); (2) A CONSORT-style study flow chart has been added as Figure 1 in the revised manuscript, detailing the patient screening, inclusion/exclusion, PSM, and group allocation process (240 screened → 240 eligible → 216 matched via PSM → 80 per group for final analysis); (3) A bar graph with error bars (mean ± SD) has been added as Figure 2, presenting the dynamic changes of Scr, BUN, and eGFR in the three groups at baseline, 24h, 48h, and 72h post-contrast for intuitive visualization; (4) Table 1 (baseline measurement data) and Table 2 (baseline count data) have been merged into a single Table 1: Baseline Characteristics of the Three Groups for conciseness; (5) F, t, χ², and Fisher’s exact values have been removed from all table columns (only P values are retained) to simplify tables; (6) All tables have been supplemented with detailed footnotes specifying measurement units, statistical tests, and abbreviations (e.g., "Data are presented as mean ± SD or n (%). Statistical tests: One-way ANOVA for continuous variables, Pearson’s χ² test for categorical variables. SMD=standardized mean difference"); (7) Stratified analysis of CIN incidence by age group (<60/≥60 years), sex, diabetes, hypertension, coronary heart disease, and NYHA class has been added as Supplementary Table 1; (8) Binary logistic regression results have been reorganized into a principal Table 6 (instead of narrative text), including the independent variable, OR, 95% CI, and P value, with the control group as the reference; (9) Tables 3, 4, and 5 (repeated-measures ANOVA, intragroup, and intergroup renal function comparisons) have been moved to Supplementary Materials to streamline the main manuscript; (10) The n=60 error in Table 7 has been corrected to n=80 (see Reviewer #1, Comment 7).

6. Discuss CIN incidence difference (observed 15% vs. introduced 30%–50%), add limitations and perspectives

Response: (1) The difference between the observed CIN incidence (15% in control group) and the 30%–50% incidence reported in the Introduction (for high-risk cohorts) has been discussed in 4.4 Interpretation of Relatively High CIN Incidence in the Control Group section: The 30%–50% incidence refers to severe high-risk cohorts (e.g., eGFR <60 mL/min with combined diabetes/hypertension, or post-cardiac angiography with high contrast dosage), while our control group included latent high-risk patients with normal eGFR (37.5% hypertension, 25% diabetes), leading to a moderate CIN incidence (15%) between low-risk and severe high-risk populations; (2) A dedicated 4.5 Study Limitations section has been added, clearly stating the retrospective design, single-center setting, small number of CIN events, short follow-up (72h), and exclusion of severe renal/cardiac dysfunction patients; (3) A 4.6 Clinical Implications and Perspectives section has been added, outlining the clinical recommendations from the study and proposing future research directions: large-sample, multicenter, prospective RCTs with long-term follow-up (6–12 months) to evaluate long-term renal outcomes, and subgroup studies in severe high-risk populations (eGFR <60 mL/min).

7. Correct typing errors (Diabetes mellitus → Diabetes, xˉ±s, Chinese symbols)

Response: All typing errors have been fully corrected: (1) "Diabetes mellitus" has been abbreviated to Diabetes throughout the manuscript for consistency; (2) The formatting error of xˉ±s has been corrected in all tables and text; (3) All accidental Chinese symbols (e.g., "组") have been deleted from the manuscript (including tables and supplementary materials). A full language and formatting review has been conducted to ensure no other errors.

Reviewer #3

1. Inconsistency of informed consent in retrospective study

Response: We apologize for the previous ambiguous description and have fully clarified the informed consent process in the Ethical Compliance section (see detailed response to Reviewer #1, Comment 2). This is a retrospective study; written informed consent was obtained retrospectively from all follow-upable patients before their data were included, and informed consent was waived for lost-to-follow-up patients with the formal approval of the Institutional Ethics Committee. All data were anonymized to comply with ethical norms, and the revised description eliminates the inconsistency.

2. Selection bias from non-randomization (group allocation based on operator opinion)

Response: We have clarified in 2.1.3 Grouping and Sample Size Justificat

Attachment Submitted filename: Response to Reviewers.docx

# Peer review: Decision Letter 1

**DOI:** 10.1371/journal.pone.0353461.r003

**Authors:** Li Yang

9 Jun 2026

PONE-D-26-01652R1

Sodium Bicarbonate versus N-Acetylcysteine plus Hydration vs Hydration Alone for Preventing Contrast-Associated Acute Kidney Injury: A Single-Center Retrospective Analysis with Propensity Score Matching

PLOS One

Dear Dr. Ma,

Thank you for submitting your manuscript to PLOS ONE. After careful consideration, we feel that it has merit but does not fully meet PLOS ONE’s publication criteria as it currently stands. Therefore, we invite you to submit a revised version of the manuscript that addresses the points raised during the review process.

Please submit your revised manuscript by Jul 24 2026 11:59PM. If you will need more time than this to complete your revisions, please reply to this message or contact the journal office at plosone@plos.org. When you're ready to submit your revision, log on to https://www.editorialmanager.com/pone/ and select the 'Submissions Needing Revision' folder to locate your manuscript file.

Please include the following items when submitting your revised manuscript:

- A letter that responds to each point raised by the academic editor and reviewer(s). You should upload this letter as a separate file labeled 'Response to Reviewers'.
- A marked-up copy of your manuscript that highlights changes made to the original version. You should upload this as a separate file labeled 'Revised Manuscript with Track Changes'.
- An unmarked version of your revised paper without tracked changes. You should upload this as a separate file labeled 'Manuscript'.

If you would like to make changes to your financial disclosure, please include your updated statement in your cover letter. Guidelines for resubmitting your figure files are available below the reviewer comments at the end of this letter.

If applicable, we recommend that you deposit your laboratory protocols in protocols.io to enhance the reproducibility of your results. Protocols.io assigns your protocol its own identifier (DOI) so that it can be cited independently in the future. For instructions see: https://journals.plos.org/plosone/s/submission-guidelines#loc-laboratory-protocols. Additionally, PLOS ONE offers an option for publishing peer-reviewed Lab Protocol articles, which describe protocols hosted on protocols.io. Read more information on sharing protocols at https://plos.org/protocols?utm_medium=editorial-email&utm_source=authorletters&utm_campaign=protocols.

As the corresponding author, your ORCID iD is verified in the submission system and will appear in the published article. PLOS supports the use of ORCID, and we encourage all coauthors to register for an ORCID iD and use it as well. Please encourage your coauthors to verify their ORCID iD within the submission system before final acceptance, as unverified ORCID iDs will not appear in the published article. *Only*  the individual author can complete the verification step; PLOS staff *cannot*  verify ORCID iDs on behalf of authors.

We look forward to receiving your revised manuscript.

Kind regards,

Li Yang, M.D.

Academic Editor

PLOS One

**Journal Requirements:**

If the reviewer comments include a recommendation to cite specific previously published works, please review and evaluate these publications to determine whether they are relevant and should be cited. There is no requirement to cite these works unless the editor has indicated otherwise.

[Note: HTML markup is below. Please do not edit.]

Reviewers' comments:

Reviewer's Responses to Questions

**Comments to the Author**

1. If the authors have adequately addressed your comments raised in a previous round of review and you feel that this manuscript is now acceptable for publication, you may indicate that here to bypass the “Comments to the Author” section, enter your conflict of interest statement in the “Confidential to Editor” section, and submit your "Accept" recommendation.

Reviewer #1: All comments have been addressed

Reviewer #2: (No Response)

Reviewer #3: All comments have been addressed

**********

2. Is the manuscript technically sound, and do the data support the conclusions?

The manuscript must describe a technically sound piece of scientific research with data that supports the conclusions. Experiments must have been conducted rigorously, with appropriate controls, replication, and sample sizes. The conclusions must be drawn appropriately based on the data presented.

Reviewer #1: Yes

Reviewer #2: Yes

Reviewer #3: No

**********

3. Has the statistical analysis been performed appropriately and rigorously?

Reviewer #1: Yes

Reviewer #2: Yes

Reviewer #3: Yes

**********

4. Have the authors made all data underlying the findings in their manuscript fully available?

The PLOS Data policy requires authors to make all data underlying the findings described in their manuscript fully available without restriction, with rare exception (please refer to the Data Availability Statement in the manuscript PDF file). The data should be provided as part of the manuscript or its supporting information, or deposited to a public repository. For example, in addition to summary statistics, the data points behind means, medians and variance measures should be available. If there are restrictions on publicly sharing data—e.g. participant privacy or use of data from a third party—those must be specified.

Reviewer #1: Yes

Reviewer #2: Yes

Reviewer #3: No

**********

5. Is the manuscript presented in an intelligible fashion and written in standard English?

PLOS ONE does not copyedit accepted manuscripts, so the language in submitted articles must be clear, correct, and unambiguous. Any typographical or grammatical errors should be corrected at revision, so please note any specific errors here.

Reviewer #1: Yes

Reviewer #2: Yes

Reviewer #3: Yes

**********

6. Review Comments to the Author

Please use the space provided to explain your answers to the questions above. You may also include additional comments for the author, including concerns about dual publication, research ethics, or publication ethics. (Please upload your review as an attachment if it exceeds 20,000 characters)

**Reviewer #1:** (No Response)

**Reviewer #2:** The authors made changes improving considerably their manuscript. However, we still have the following suggestions:

- Please add to section "2.6 Sample Size Calculation" the comments placed in Point-by-Point Responses to Reviewers’ Comments, specifically the power size recalculation.

- Add footnotes to Table 2.

- Figure 2 and Supplementary information is mentioned in Responses to Reviewers but not localized in the current version.

**Reviewer #3:**  Solutions for removing of confounding factors have not been addressed clearly, especially about selection biases and other confounders

**********

7. PLOS authors have the option to publish the peer review history of their article (what does this mean?). If published, this will include your full peer review and any attached files.

If you choose “no”, your identity will remain anonymous but your review may still be made public.

**Do you want your identity to be public for this peer review?**  For information about this choice, including consent withdrawal, please see our Privacy Policy.

Reviewer #1: **Yes:** Abdul Rehman Arshad

Reviewer #2: No

Reviewer #3: **Yes:** Hassan Argani

**********

[NOTE: If reviewer comments were submitted as an attachment file, they will be attached to this email and accessible via the submission site. Please log into your account, locate the manuscript record, and check for the action link "View Attachments". If this link does not appear, there are no attachment files.]

To ensure your figures meet our technical requirements, please review our figure guidelines: https://journals.plos.org/plosone/s/figures

You may also use PLOS’s free figure tool, NAAS, to help you prepare publication quality figures: https://journals.plos.org/plosone/s/figures#loc-tools-for-figure-preparation.

NAAS will assess whether your figures meet our technical requirements by comparing each figure against our figure specifications.

# Author comment: Author response to Decision Letter 2

**DOI:** 10.1371/journal.pone.0353461.r004

15 Jun 2026

Response to Editorial Instructions

Manuscript ID: PONE-D-26-01652R1

Title: Sodium Bicarbonate versus N-Acetylcysteine plus Hydration vs Hydration Alone for Preventing Contrast-Associated Acute Kidney Injury: A Single-Center Retrospective Analysis with Propensity Score Matching

Dear Dr. Li Yang and the esteemed reviewers,

Thank you very much for your careful and constructive comments on our manuscript. We have carefully addressed all the points raised and revised the manuscript accordingly. All changes are highlighted using track changes in the "Revised Manuscript with Track Changes" file, and a clean version is provided as "Manuscript". Below is our point-by-point response to each comment.

We believe the revised manuscript has been significantly improved and now fully meets the publication criteria of PLOS ONE. We look forward to your favorable consideration.

Sincerely, Dr. Ying-Huan Ma and co-authors Corresponding Author Department of Nephrology, The Air Force Hospital of Northern Theater PLA Email: 952006555@qq.com

Response to Reviewers

Response to Reviewer #1

Comment: All comments have been addressed.

Response: We sincerely appreciate your recognition and support of our work. We have carefully reviewed the entire manuscript again to ensure no typos or formatting errors remain, and all previous revisions are retained in the current version.

Response to Reviewer #2

Comment 1: Please add to section "2.6 Sample Size Calculation" the comments placed in Point-by-Point Responses to Reviewers’ Comments, specifically the power size recalculation.

Response: Thank you for this important suggestion. We have added detailed post-hoc power analysis results to Section 2.6 Sample Size Calculation, including the statistical power for the primary outcome and subgroup analyses, as well as the rationale for not including an attrition rate. The revised section is provided below (see Manuscript, Section 2.6, Lines 265–289).

Comment 2: Add footnotes to Table 2.

Response: We have added comprehensive footnotes to Table 2, explaining the Greenhouse-Geisser correction, the definition of partial η² (effect size), and the interpretation of effect size magnitudes. The revised Table 2 is included in the manuscript (see Manuscript, Table 2, Lines 484–488).

Comment 3: Figure 2 and Supplementary information is mentioned in Responses to Reviewers but not localized in the current version.

Response: We apologize for this oversight. We have:Added Figure 2 (Dynamic changes in serum creatinine and eGFR over 72 hours in the three groups) to the Results section, with corresponding citations in the text (see Manuscript, Section 3.2.2, Lines489–509).

Response to Reviewer #3

Comment: Solutions for removing of confounding factors have not been addressed clearly, especially about selection biases and other confounders.

Response: Thank you for this critical comment. We have comprehensively revised the manuscript to clarify our strategies for controlling confounding factors and selection bias, with revisions in three key sections:

1.Section 2.4 Group Allocation (Lines 228–244): Added detailed explanation of how our institutional protocol-based grouping minimized physician-related selection bias, and why we included all core baseline risk factors in the PSM model to address residual confounding from risk stratification.

2.Section 2.5 Propensity Score Matching (Lines 245–264): Expanded the description of PSM methodology, including the rationale for choosing 1:1:1 nearest-neighbor matching with a 0.05 caliper, the use of standardized mean difference (SMD) for balance assessment, and verification of the common support domain.

3.Section 4.6 Study Limitations (Lines 709–749): Explicitly acknowledged the potential for residual confounding from unmeasured variables (e.g., baseline volume status, contrast injection rate) and explained how our rigorous study design and PSM adjustment minimized this risk.

All revisions are highlighted in the track changes version, and we believe they fully address your concerns about confounding and selection bias.

Attachment Submitted filename: Response_to_Reviewers_auresp_2.docx

# Peer review: Decision Letter 2

**DOI:** 10.1371/journal.pone.0353461.r005

**Authors:** Li Yang

24 Jun 2026

Sodium Bicarbonate versus N-Acetylcysteine plus Hydration vs Hydration Alone for Preventing Contrast-Associated Acute Kidney Injury: A Single-Center Retrospective Analysis with Propensity Score Matching

PONE-D-26-01652R2

Dear Dr. Ma,

We’re pleased to inform you that your manuscript has been judged scientifically suitable for publication and will be formally accepted for publication once it meets all outstanding technical requirements.

Within one week, you’ll receive an e-mail detailing the required amendments. When these have been addressed, you’ll receive a formal acceptance letter and your manuscript will be scheduled for publication.

An invoice will be generated when your article is formally accepted. Please note, if your institution has a publishing partnership with PLOS and your article meets the relevant criteria, all or part of your publication costs will be covered. Please make sure your user information is up-to-date by logging into Editorial Manager at Editorial Manager® and clicking the ‘Update My Information' link at the top of the page. For questions related to billing, please contact billing support.

If your institution or institutions have a press office, please notify them about your upcoming paper to help maximize its impact. If they’ll be preparing press materials, please inform our press team as soon as possible -- no later than 48 hours after receiving the formal acceptance. Your manuscript will remain under strict press embargo until 2 pm Eastern Time on the date of publication. For more information, please contact onepress@plos.org.

Kind regards,

Li Yang, M.D.

Academic Editor

PLOS One

Additional Editor Comments (optional):

Reviewers' comments:

Reviewer's Responses to Questions

**Comments to the Author**

1. If the authors have adequately addressed your comments raised in a previous round of review and you feel that this manuscript is now acceptable for publication, you may indicate that here to bypass the “Comments to the Author” section, enter your conflict of interest statement in the “Confidential to Editor” section, and submit your "Accept" recommendation.

Reviewer #2: All comments have been addressed

Reviewer #3: All comments have been addressed

**********

2. Is the manuscript technically sound, and do the data support the conclusions?

The manuscript must describe a technically sound piece of scientific research with data that supports the conclusions. Experiments must have been conducted rigorously, with appropriate controls, replication, and sample sizes. The conclusions must be drawn appropriately based on the data presented.

Reviewer #2: Yes

Reviewer #3: Yes

**********

3. Has the statistical analysis been performed appropriately and rigorously?

Reviewer #2: Yes

Reviewer #3: Yes

**********

4. Have the authors made all data underlying the findings in their manuscript fully available?

The PLOS Data policy requires authors to make all data underlying the findings described in their manuscript fully available without restriction, with rare exception (please refer to the Data Availability Statement in the manuscript PDF file). The data should be provided as part of the manuscript or its supporting information, or deposited to a public repository. For example, in addition to summary statistics, the data points behind means, medians and variance measures should be available. If there are restrictions on publicly sharing data—e.g. participant privacy or use of data from a third party—those must be specified.

Reviewer #2: Yes

Reviewer #3: Yes

**********

5. Is the manuscript presented in an intelligible fashion and written in standard English?

PLOS ONE does not copyedit accepted manuscripts, so the language in submitted articles must be clear, correct, and unambiguous. Any typographical or grammatical errors should be corrected at revision, so please note any specific errors here.

Reviewer #2: Yes

Reviewer #3: Yes

**********

6. Review Comments to the Author

Please use the space provided to explain your answers to the questions above. You may also include additional comments for the author, including concerns about dual publication, research ethics, or publication ethics. (Please upload your review as an attachment if it exceeds 20,000 characters)

Reviewer #2: Thank you for considering me as a reviewer. The authors had made all the requested changes in their manuscript.

Reviewer #3: Dear author,

Thank you for your revised manuscript. Your manuscript is free of ethic problems. Although this retrospective study has been exposed inevitably to a couple of unintentional confounding factors, almost all of my requested items have been addressed appropriately.

**********

7. PLOS authors have the option to publish the peer review history of their article (what does this mean?). If published, this will include your full peer review and any attached files.

If you choose “no”, your identity will remain anonymous but your review may still be made public.

**Do you want your identity to be public for this peer review?**  For information about this choice, including consent withdrawal, please see our Privacy Policy.

Reviewer #2: No

Reviewer #3: **Yes:** Hassan Argani

**********

# Editor report: Acceptance letter

**DOI:** 10.1371/journal.pone.0353461.r006

**Authors:** Li Yang

PONE-D-26-01652R2

PLOS One

Dear Dr. Ma,

I'm pleased to inform you that your manuscript has been deemed suitable for publication in PLOS One. Congratulations! Your manuscript is now being handed over to our production team.

At this stage, our production department will prepare your paper for publication. This includes ensuring the following:

* All references, tables, and figures are properly cited

* All relevant supporting information is included in the manuscript submission,

* There are no issues that prevent the paper from being properly typeset

You will receive further instructions from the production team, including instructions on how to review your proof when it is ready. Please keep in mind that we are working through a large volume of accepted articles, so please give us a few days to review your paper and let you know the next and final steps.

Lastly, if your institution or institutions have a press office, please let them know about your upcoming paper now to help maximize its impact. If they'll be preparing press materials, please inform our press team within the next 48 hours. Your manuscript will remain under strict press embargo until 2 pm Eastern Time on the date of publication. For more information, please contact onepress@plos.org.

You will receive an invoice from PLOS for your publication fee after your manuscript has reached the completed accept phase. If you receive an email requesting payment before acceptance or for any other service, this may be a phishing scheme. Learn how to identify phishing emails and protect your accounts at https://explore.plos.org/phishing.

If we can help with anything else, please email us at customercare@plos.org.

Thank you for submitting your work to PLOS ONE and supporting open access.

Kind regards,

PLOS ONE Editorial Office Staff

on behalf of

Dr. Li Yang

Academic Editor

PLOS One
